# No Reproducibility, No Progress: Rethinking CT Benchmarking

**DOI:** 10.3390/jimaging11100344

**Published:** 2025-10-02

**Authors:** Dmitry Polevoy, Danil Kazimirov, Marat Gilmanov, Dmitry Nikolaev

**Affiliations:** 1 Federal Research Center Computer Science and Control RAS, 119333 Moscow, Russia; 2 Smart Engines Service LLC., 117312 Moscow, Russia; 3 Institute for Information Transmission Problems RAS, 127051 Moscow, Russia

**Keywords:** computed tomography (CT), X-ray CT (xCT), virtual CT (vCT), benchmarking, reproducibility, data preparation, quality assessment, evaluation metrics, machine learning (ML), deep learning (DL), datasets, phantoms, CT reconstruction methods

## Abstract

Reproducibility is a cornerstone of scientific progress, yet in X-ray computed tomography (CT) reconstruction, it remains a critical and unresolved challenge. Current benchmarking practices in CT are hampered by the scarcity of openly available datasets, the incomplete or task-specific nature of existing resources, and the lack of transparent implementations of widely used methods and evaluation metrics. As a result, even the fundamental property of reproducibility is frequently violated, undermining objective comparison and slowing methodological progress. In this work, we analyze the systemic limitations of current CT benchmarking, drawing parallels with broader reproducibility issues across scientific domains. We propose an extended data model and formalized schemes for data preparation and quality assessment, designed to improve reproducibility and broaden the applicability of CT datasets across multiple tasks. Building on these schemes, we introduce checklists for dataset construction and quality assessment, offering a foundation for reliable and reproducible benchmarking pipelines. A key aspect of our recommendations is the integration of virtual CT (vCT), which provides highly realistic data and analytically computable phantoms, yet remains underutilized despite its potential to overcome many current barriers. Our work represents a first step toward a methodological framework for reproducible benchmarking in CT. This framework aims to enable transparent, rigorous, and comparable evaluation of reconstruction methods, ultimately supporting their reliable adoption in clinical and industrial applications.

## 1. Introduction

### 1.1. Modern Challenges of Reproducibility and Benchmarking in CT Reconstruction

Modern scientific research increasingly relies on the development of new methods for analyzing and processing domain-specific data. To ensure objective comparison of such methods, benchmarking—systematic evaluation on predefined datasets with relevant quality metrics—has become essential [1]. However, the reliability of benchmarking critically depends not only on carefully chosen datasets and appropriate metrics, but also on strict adherence to reproducible research principles [1,2].

Open access to data and code is pivotal for benchmarking, enabling verification of results and fostering innovation [1]. Despite these benefits, many curated datasets remain inaccessible, leading to suboptimal or inconsistent evaluation practices. A frequent issue is the “off-label” use of data, when datasets designed for one purpose are applied to another, often undermining the validity of conclusions and making experiments difficult to reproduce [3]. Similarly, inadequately chosen metrics can distort results and reduce research validity, particularly in image processing tasks. International machine learning (ML) and deep learning (DL) competitions often reveal issues with task categorization and metric selection [4,5]. The outlined problems illustrate a broader concern: without transparent datasets, well-justified metrics, and explicit documentation of experimental procedures, even widely cited results may lack reproducibility and fail to provide a reliable basis for further research.

In this context, special attention must be paid to the concept of *reproducibility*, which we emphasize as a central requirement for benchmarking and training pipelines. According to established definitions [6], three related but distinct notions must be separated. *Repeatability* refers to obtaining the same results when the same research team repeats an experiment under identical conditions, ensuring stability of the implementation and experimental setup. *Reproducibility*, in the narrower sense, denotes achieving consistent results when certain conditions are varied, such as changes in software libraries, hardware, or random seeds, while the core methodology and data remain the same. *Replicability* goes one step further and concerns whether different teams, often using different implementations, datasets, or even experimental designs, can arrive at consistent scientific conclusions. For benchmarking purposes, reproducibility is the most suitable criterion because it balances rigor and practicality: it ensures that method performance is stable under minor variations that naturally occur across runs, software environments, and hardware, providing a reliable and fair comparison of methods. However, notwithstanding its apparent importance, reproducibility frequently breaks down due to insufficient documentation of preprocessing and dataset splits, hidden sources of nondeterminism, or variability (e.g., random seeds, library versions, or hardware variability), environment drift, and methodological flaws such as data leakage [6,7,8].

Across scientific domains, systemic barriers to reproducibility include the lack of standardized datasets and evaluation protocols, variability induced by hardware and compilers, under-reporting of hyperparameters, and stochastic training dynamics [7]. Procedural drivers that improve reproducibility include adherence to FAIR/TOP principles, structured reporting artifacts such as data and model cards, multi-seed experiments, and testing on multiple datasets to counter optimistic results [7]. From a principled perspective, reproducibility requires faithful re-execution of a documented scientific method—covering data, procedures, implementation, environment, analysis, and interpretation—with artifacts and conditions separated clearly to enable independent verification [6].

The reproducibility-related issues are especially pertinent in X-ray computed tomography (X-ray CT, or xCT) [9], a non-destructive imaging method that reconstructs the internal structure of an object from projection images [10]. Reproducibility-related challenges in CT arise at every stage of benchmarking, from the limited availability of open-access datasets—which typically include projection data (sinograms) and ground truth (GT) reconstructions—to unpublished or poorly documented implementations of the studied methods and examined quality assessment functions. All of these hinder objective and reliable evaluation, particularly in light of the rise of ML- and DL-based reconstruction methods [11].

In medical imaging, and specifically in DL-based CT reconstruction, reproducibility is particularly difficult to achieve. Challenges include small and heterogeneous cohorts, variability across imaging modalities and organs, inter- and intra-operator annotation inconsistency, and sensitivity to preprocessing and hyperparameters [12]. These issues make it difficult to reproduce published pipelines even when source code is provided. Furthermore, reliance on private or proprietary datasets limits independent verification, contributing to the reproducibility crisis in medical AI research [7,8].

In industrial CT, reproducibility challenges manifest differently. Here, factors such as variability in acquisition hardware, calibration, and proprietary reconstruction algorithms introduce difficulties in reproducing results across facilities [7]. While datasets may be larger than in medical imaging, restrictions on data sharing due to confidentiality and intellectual property concerns remain major barriers.

Virtual CT (vCT) has emerged as an attractive alternative to physical measurements, as it enables flexible experimental setups and often replicates workflows designed for real data. Yet, reproducibility in vCT-based research is far from guaranteed. Due to the scarcity of reliable raw sinograms, many studies rely on postprocessed reprojections from existing reconstructions or entirely synthetic data. While these resources can facilitate rapid prototyping, they often yield overly optimistic performance estimates that fail to transfer to real-world conditions. An additional source of bias stems from the definition of GT: in practice, GTs are frequently chosen as the highest-quality reconstruction available, sometimes generated by proprietary algorithms. Such GTs may inadvertently embed method-specific artifacts, encouraging ML and DL models to reproduce these artifacts rather than approximate the true object structure, thereby reducing external validity and limiting reproducibility across datasets.

The selection of evaluation metrics further complicates the situation. Metrics must not only quantify pixel-wise similarity but also capture clinically or industrially relevant aspects of reconstruction quality. Inappropriate or overly narrow metrics can yield misleading conclusions about model performance and practical utility. Combined with opaque GT definitions and synthetic data usage, this creates benchmarking pipelines that lack reproducibility and, consequently, fail to provide a reliable basis for methodological progress.

En route towards reproducibility, improving CT benchmarking therefore requires a threefold effort: (i) establishing openly accessible datasets that include both raw projection data and validated GT volumes; (ii) ensuring transparent documentation of preprocessing, reconstruction parameters, and evaluation procedures; and (iii) critically revisiting metric design to incorporate task-specific requirements. Only under these conditions can the CT field achieve reproducible, objective comparisons of methods and ensure that advances in reconstruction techniques translate into reliable clinical and industrial applications.

### 1.2. Contribution

This work makes the following key contributions to advancing reproducibility and benchmarking in CT reconstruction:

1. **Critical analysis of current practices.** We systematically reviewed the state of benchmarking in CT, identifying systemic shortcomings, including the scarcity of open datasets, inadequate or opaque documentation, reliance on non-transparent ground truth definitions, and inconsistent use of evaluation metrics. These issues collectively undermine reproducibility and hinder methodological progress.
2. **Formalization of benchmarking workflows.** We introduced a *data model* and formally described the *data preparation* and *quality assessment schemes* commonly used in practice. This formalization provides a structured view of benchmarking pipelines, clarifying hidden assumptions and sources of variability that have previously limited reproducibility.
3. **Extension of existing dataset preparation schemes.** Building on this formalization, we proposed *extended schemes* that support dataset preparation across a wider range of tasks and domains. The extended schemes explicitly incorporate reproducibility as a guiding principle, enabling more reliable comparisons of CT reconstruction methods under diverse experimental conditions.
4. **Checklist for reproducible benchmarking.** We formulated a practical *checklist* for dataset curation, quality evaluation, and benchmarking procedures. This checklist emphasizes transparency, standardization, and reproducibility, drawing on established practices from ML and DL research. Importantly, we highlight the underutilized potential of *virtual CT (vCT)* with analytically computable phantoms as a robust source of realistic and fully controllable data.
5. **Path toward standardization.** Taken together, these contributions lay the groundwork for the establishment of a *standardized, reproducible benchmarking framework* in CT. By aligning benchmarking practices in CT with those already well-developed in other scientific domains, this work advances the methodological rigor of CT research and supports the development of trustworthy, generalizable reconstruction methods for both clinical and industrial applications.

### 1.3. Paper Organization

This paper is organized as follows. Section 1 provides an introductory overview of the current state of benchmarking in CT research, with particular emphasis on existing challenges, especially those related to reproducibility. The main contributions are summarized in Section 1.2. Section 2 introduces the general methodological concepts and terminology used throughout this paper. Section 3 reviews and analyzes current techniques for dataset preparation, as well as approaches to measuring and comparing quality and performance in CT tasks. A consolidated table of publicly available volumetric vCT datasets for benchmarking is presented within Section 3.3. Section 4 discusses in detail the flaws of existing datasets and benchmarks. Section 5 introduces a data model for dataset preparation and benchmarking, along with a formalization of the most widely adopted modeling and quality assessment schemes, analyzing their specific properties. Section 6 describes our extended schemes for data modeling and quality assessment, designed to achieve high reproducibility. Section 6.3 further provides a checklist of proposals for practices in CT dataset and benchmark creation conducive to reproducibility, which can be directly applied to dataset preparation, quality assessment, and comprehensive method comparison. Section 7 outlines future directions toward the standardization of data preparation, evaluation protocols, and the establishment of robust and reproducible CT benchmarking. Finally, Section 8 concludes this paper.

## 2. Context and Terms

### 2.1. X-Ray CT Reconstruction

In the following, we focus, without loss of generality, on xCT and the specific aspects of xCT data modeling. Methodologically, the benchmarking challenges discussed and the approaches proposed to address them are relevant to a wide range of CT modalities.

The primary objective of the CT is to reconstruct the spatial distribution of the X-ray linear attenuation coefficient (LAC), μ(x,e), $x\in R^{3}$, where *e* is an energy value, using the measured projection data, or sinograms, of the object under investigation [13,14]. The result of a CT reconstruction is referred to as the reconstruction or reconstructed volume. Visualization of the reconstruction enables the assessment of the spatial distribution of the X-ray LAC within the object.

In medical imaging, CT reconstruction results are typically reported and stored in Hounsfield Units (HU) rather than in LAC. The formula for determining attenuation parameters in HU [15] for a given energy *e* of a monochromatic spectrum is expressed as follows:

(1) $$HU_{air}=1000\cdot \frac{\mu_{water}-\mu_{material}}{\mu_{water}-\mu_{air}},$$

where $\mu_{material}$ denotes the LAC of the material composing the object under investigation, while $\mu_{water}$ and $\mu_{air}$ correspond to the LACs of water and air at a given energy *e*, respectively. The LAC of air is commonly assumed to be approximately zero in the literature [16]. This assumption leads to the following simplified formula:

(2) $$HU_{vac}=1000\cdot \frac{\mu_{water}-\mu_{material}}{\mu_{water}}.$$

The task of improving reconstruction quality is often formulated as the suppression of the reconstruction artifacts of specific types and origins. In the context of CT, several sub-tasks aim to enhance reconstruction quality by addressing the following:

1. Beam-hardening artifacts (BH artifacts): suppression algorithms, referred to as beam-hardening correction (BHC) methods, are employed to improve the quality of polychromatic data reconstruction within the monochromatic model [17];
2. Metal-like artifacts: these are caused by highly attenuating inclusions [18,19];
3. Scatter artifacts: resulting from the scattering of X-rays [20];
4. Low-dose artifacts: occurring when the radiation dose is reduced [21];
5. Denoising: denoising task in CT involves processing either raw projection data (sinograms) or reconstructed volumes to reduce noise [22];
6. Sparse-angle (sparse-view, or few-view) artifacts: observed when reconstructing from a sparse array of projection data [23];
7. Limited-angle artifacts: occurring with a restricted angular scanning range [24];
8. Non-uniform angular range artifacts: arising from uneven angular scanning steps, as seen in monitored (anytime) CT [25,26,27];
9. Ring artifacts [28]: typically caused by miscalibrated detector cells;
10. Geometry artifacts: resulting from geometric miscalibration of the source-detector system [29,30];
11. Motion artifacts: including specific cases such as sample vibration and jitter during measurement [31].

Enhancing reconstruction quality, considering the sub-tasks above, can be achieved through (similar to the categorization for denoising methods outlined in reference [32]):

- Preprocessing (projection-domain, sinogram-domain): this involves additional processing of sinograms before reconstruction to reduce the presence and severity of artifacts in the final reconstruction;
- Postprocessing (reconstruction-domain, image-domain): processing the already reconstructed dataset to mitigate artifacts;
- Task-oriented reconstruction methods (dual-domain): combining sinogram preprocessing and volume postprocessing.

Different approaches to developing reconstruction quality improvement methods require varying real-world data for testing and optimization. For preprocessing-based methods, raw sinograms are essential for testing their performance. Conversely, postprocessing methods require reconstructed volumes representing the internal structure of test objects.

### 2.2. The Consumer Landscape of CT

Let us consider the main application areas in terms of consumer demands and the potential uses of xCT results. The most extensively represented in scientific publications and open datasets is medical CT (medCT). MedCT scanning is a widely used diagnostic method. CT may be prescribed either as a single examination or repeatedly to monitor disease progression (for example, in the treatment of oncological or severe respiratory conditions). The primary consumers of CT reconstruction results are radiologists; therefore, researchers and developers aim to produce visually standardized images that, regardless of the method of acquisition, are suitable for expert diagnostic evaluation. In medical practice, CT scanning is applied to relatively homogeneous biological objects, characterized by limited variability in X-ray material densities (tissues). The presence of highly absorbing inclusions constitutes a deviation from normal imaging conditions, and the correction of resulting artifacts is regarded as an important, independent task. Medical protocols regulate all aspects of image acquisition, including patient positioning, scanning modes, reconstruction and normalization algorithm parameters, and related factors. Particular emphasis is placed on the development of safer methods—specifically, reducing radiation dose while preserving diagnostic image quality.

From the perspective of open datasets and benchmarks in medicine, the situation remains ambiguous. On the one hand, the development of ML/DL methods for the analysis of reconstructions has stimulated the creation and public release of large volumes of medical imaging data, along with associated medical and technical information, including GT for segmentation and detection tasks [33]. On the other hand, access to raw data/sinograms is restricted to a small number of research groups affiliated with major medical equipment manufacturers, many of which occupy monopolistic or near-monopolistic positions in the market.

There is no established definition of the term industrial CT (indCT), and in this study, we use it to denote all non-medical applications of xCT, which we conditionally divide into laboratory CT (labCT) and conveyor CT (conCT).

We use the term labCT to refer to studies in which researchers control all aspects of data acquisition and processing and have full access to the data. Such studies most often involve unique experiments, where the objects of investigation, the equipment, and/or the scanning conditions are not reproducible. In some cases, the best reconstruction results are published, but without the corresponding projection data.

We also classify as labCT the studies conducted by groups directly engaged in the development of CT methods. The datasets they publish, even when projection data are included, are characterized by the uniqueness of the CT setups and by the limited diversity of objects and scanning conditions.

The term conCT is defined as industrial applications of xCT with a fixed data-processing conveyor. ConCT tasks feature a clear separation between the setup/commissioning phase and the operational phase. The first phase resembles labCT in terms of optimizing acquisition parameters, reconstruction pipelines, and subsequent data processing. The second phase is similar to medCT, as mass scanning and analysis of relatively uniform industrial objects are performed within a limited set of operating modes to address non-destructive testing (NDT) and related tasks. An example of characteristic features of conCT is the focus on minimizing scanning time through a reduction in the number of projections. Although successful pipelines for vCT data generation have been described [9,11], there are currently no published datasets obtained from real industrial conveyor systems or vCT simulations.

### 2.3. Repeatability, Reproducibility and Replicability

In recent years, there has been growing attention within the ML/DL community to the issues of repeatability, reproducibility, and replicability in scientific research. Comprehensive accounts of accomplishments, the present state, outstanding challenges, and relevant references to prior work are provided in recent literature [7,8]. Most of the advances in this area are readily applicable to research in CT. In the following analysis, we will use an extended version of the types of validation studies (see Table 1) from the reproducibility framework [8].

The greatest attention to reproducibility issues in CT publications is observed in medical studies, where ML/DL methods are applied to CT reconstruction results. Large volumes of annotated data are being accumulated, along with positive experience in standardizing metadata and GT annotations [33,34,35]. Another important example in medicine is standardized quantitative radiomics [36,37,38], which represents a successful case of systematic efforts to enhance reproducibility, as well as the development of robust and reliable analysis methods that account for variability in CT images.

Next, we review publicly available CT (vCT) datasets and benchmarks specifically for CT reconstruction.

## 3. Open Sources Review

### 3.1. Datasets

There is a significant scarcity of publicly available and versatile datasets for many of the aforementioned sub-tasks (see list in SubSection 2.1). For some challenges, such datasets are entirely absent. For instance, most low-dose CT (LDCT) methods are trained and evaluated on datasets provided through the 2016 NIH-AAPM-Mayo Clinic LDCT Grand Challenge [39], or the subsequently released, larger dataset, LDCT and Projection data [40], which features a greater number of images and broader anatomical coverage.

Another notable dataset is the publicly available 2DeteCT dataset [41], which offers paired projections and reconstructions for low-dose reconstruction (LDR) and BHC tasks and stands as the only known publicly available dataset of its kind. This dataset includes scans (collected under various imaging conditions) of non-medical objects and proposes to use reconstructions obtained from projections measured with a monochromatic source as the GT.

For other tasks targeting specific artifact suppression, there are no publicly available volumetric datasets with GT. For example, the suppression of ring artifacts [28,42,43] is supported by only one dataset of real data [44], which lacks a true GT. Similarly, no open datasets with pronounced scattering artifacts and known GT are currently available. The situation is analogous for datasets aimed at geometry correction or motion artifact suppression.

Given the limited availability of diverse real-world CT datasets, particularly those containing raw projections, vCT presents a promising direction for dataset modeling.

In the following sections, we will examine the vCT dataset generation for specific tasks.

### 3.2. Modeling Through Sinogram Modification (Without Simulating Forward Projections)

For a range of specialized formulations of the CT reconstruction and postprocessing tasks, evaluation datasets are often generated by modifying original sinograms obtained during scanning, without simulating forward projections.

For instance, a common approach to dose reduction in CT scanning simulation is based on decreasing the number of projections utilized in the reconstruction procedure. If the projections are spaced more sparsely but evenly, the problem is referred to as sparse-angle or sparse-view CT [45,46]. In limited-angle CT, not only is the number of projections reduced, but restrictions are also imposed on the scanning angles’ range [46,47]. Sinogram modeling in these cases involves selecting projections (in the desired number) that meet the angular constraints [46,48,49,50]. In the context of monitored tomographic reconstruction [25,26,51,52], both the projection angle selection strategy and the total number of projections may vary.

Another practical approach to dose reduction in CT scanning is decreasing the number of photons, achieved by reducing the X-ray tube current and/or shortening the exposure time. The increased impact of noise on reconstruction results in this case is modeled by artificially adding noise to the original sinograms to achieve the desired simulated characteristics [53,54,55,56,57].

Despite the widespread use of sinogram-noising techniques and publicly available datasets in medical research, the standard noise model is formulated under the assumption of a monochromatic source and is validated on medical data subjected to water correction. When BH effects become more pronounced, substantial discrepancies are observed between the simulation results and actual real measurements [58].

To assess the quality of reconstruction and postprocessing, FR-IQA is commonly used. GT is typically defined as the highest-quality CT reconstruction of the original sinograms, performed with all projection angles available and without additional noise.

### 3.3. vCT-Based Modeling of the Forward Projections

Let us consider the publicly available volumetric datasets obtained through vCT (Table 2).

In all datasets presented in the table, forward projections are modeled for voxel-based phantoms derived from CT images reconstructed during medical studies except AAPM TrueCT.

The modeling of a polychromatic source is achieved by integrating projections across various energies according to the source spectrum. To simulate low-dose projections, Poisson noise is applied.

The deviation of the actual polychromatic spectrum from the monochromatic model spectrum significantly affects reconstruction results, leading to discrepancies from the desired outcome. Consequently, a standard practice in medical CT scanners involves providing projections that have undergone BHC and are adjusted to an effective energy. The latter procedure is often referred to as water (water-equivalent) correction [70].

#### 3.3.1. LoDoPaB-CT

The first publicly available vCT-generated volumetric dataset was the Low-Dose Parallel Beam (LoDoPaB)-CT dataset [59], which models projections for individual slices of medical CT reconstructions using a parallel beam geometry and a linear-array detector. To prevent the inverse crime [71], bilinear interpolation is applied to upscale the slices to a higher spatial resolution before forward projection, and uniformly distributed noise in the range [0,1) is added to the original integer values in Hounsfield units (HU) for dequantization. The conversion of HU values to linear attenuation coefficients (LAC) is based on a monochromatic X-ray spectrum with an approximate energy of 60 keV. The authors adopt the common approach of using reconstructions obtained via a baseline method, such as filtered back projection (FBP) [72], from projections at normal dose levels as the GT.

#### 3.3.2. SynDeepLesion

Despite the challenges of reproducing simulations and downloading data, the pipeline proposed by Zhang et al. [61] and the SynDeepLesion dataset [63] are frequently cited in neural network-based metal artifact reduction (MAR) approaches. For a single slice in the fan-beam CT scheme, both noisy metal-free and metal-inserted sinograms are simulated. To evaluate the performance of MAR algorithms, it is recommended to use Full-Reference Image Quality Assessment (FR-IQA), where the GT is represented by metal-free CT reconstructions obtained via a reference reconstruction algorithm. In reference [64], the baseline simulation scheme [61] is extended by introducing a water correction step (BHC) for the sinogram [70]; however, neither the data generated in this manner nor the corresponding code for its generation has been made publicly available.

#### 3.3.3. AAPM Truth-Based CT (TrueCT) Reconstruction Grand Challenge

The AAPM Truth-based CT (TrueCT) Reconstruction Grand Challenge [65] was conducted to evaluate methods for medical CT reconstruction. Participants were provided with a volumetric dataset of simulated tomograms generated on the DukeSim virtual scanner using a spiral scanning scheme for 200 voxelized human models with various pathologies. The virtual scanner modeled several features of clinical CT scanners, including focal spot instability, tube current modulation, scatter protection grids, and beam hardening. To enable voxel-level comparison, CT reconstructions were resampled using linear interpolation to match the spatial resolution of the original models with isotropic voxels.

Unlike other datasets, this study defined GT as a monoenergetic representation of the original phantoms in HU at an effective energy of 64 keV. Additionally, participants were provided with sinograms and sample reconstructions of test objects (a cylindrical water phantom and an ACR phantom with multiple inserts) to validate the correctness of their reconstruction algorithms.

#### 3.3.4. AAPM CT Metal Artifact Reduction (CT-MAR) Grand Challenge

In the AAPM CT Metal Artifact Reduction (CT-MAR) Grand Challenge, participants were tasked with developing algorithms for tomographic reconstruction that compensate for artifacts caused by highly attenuating inclusions (MAR) in 2D, with a clinically representative reference provided for evaluation. The data were generated using the open-source CT simulation environment XCIST [73], employing a hybrid data simulation system that combines publicly available clinical CT images with virtual metallic objects. For simplicity, the MAR task was formulated for a fan-beam geometry, with the virtual scanner’s detector modeled as curved and rectangular in shape. This approach increases simulation time but allows for more accurate modeling of scatter effects and detector element crosstalk. The AAPM CT-MAR simulation framework includes scatter correction and BHC (water correction) of the projections; however, detailed models and parameters are available only in the form of the published code [67].

#### 3.3.5. ICASP CBCT

As part of the ICASSP-2024 3D-CBCT Challenge [68], a dataset was published to address the shortage of benchmark data for training and evaluating reconstruction methods in a cone-beam scheme under low-dose monoenergetic source conditions. Reconstruction results for low-dose sinograms were proposed to be compared with full-dose reconstructions. However, the dataset proved to be unsuitable for the intended tasks due to a range of issues [5].

### 3.4. CT Reconstruction Quality Assessment

The gold standard for assessing the quality of medical images remains Subjective Assessment Techniques (SATs), which involve expert evaluations by medical professionals. However, this approach is often prohibitively expensive and impractical for systematic benchmarking.

To evaluate various technical aspects of CT methods and equipment, numerous objective image quality metrics have been developed [74,75], including CT number accuracy and stability, contrast-to-noise ratio (CNR), noise power spectrum (NPS), and spatial resolution (often represented as PSF/LSF/ESF and MTF), among others. Most of these metrics, however, require the analysis of reconstructions of dedicated calibration phantoms with known properties. The ability to apply or interpret these metrics for comparing the results of various reconstruction methods hinges on strict assumptions about imaging system properties, such as linearity, shift invariance, or noise stationarity. For many modern iterative and learning-based reconstruction methods, these assumptions often do not hold [74], prompting the exploration of alternative metrics such as the task transfer function (TTF) or detectability index ($d^{\prime }$).

Nevertheless, a significant proportion of studies describing and analyzing specific reconstruction methods rely on simple metrics like PSNR, RMSE, SSIM, or their modifications, which often align poorly with expert assessments. More suitable and task-specific metrics for medical imaging [76,77] are continually proposed and evaluated in various publications, such as Visual Information Fidelity (VIF) [78], Fréchet Inception Distance (FID) [79], Radiomic Feature Similarity (RFS) [76], and $HaarPsi_{MED}$ [80], among others. Despite extensive research [75,76,81,82,83,84], no universally accepted objective quality assessment methodology exists that aligns well with expert evaluations across a wide range of protocols and imaging conditions.

A common research practice is to evaluate not only entire volumes but also specific regions of interest (ROIs or VOIs) of varying shapes. Depending on the target task, various metrics may be applied within these VOIs [66,75,76,83].

Most studies in the field of industrial CT (indCT) adopt the practices established in medical CT (medCT), particularly in the use of FR-IQA [75], although there are also examples of methods developed for the quantitative assessment of artifact severity in indCT images [85,86,87]. Another line of research focuses on extending benchmarking tools through techniques for evaluating geometric deviations and errors by means of:

- high-precision positioning systems for the object during scanning;
- specially manufactured physical reference objects with high geometric accuracy;
- comparison of reconstructed images with high-precision geometric descriptions of reference objects obtained using a coordinate measuring machine.

The reproducibility of such experiments is extremely limited, as they require unique and often inaccessible equipment, and no corresponding datasets have been published.

### 3.5. Benchmarks

There are no openly available and systematically used benchmarks for CT reconstruction methods. As a result, no standardized or community-wide consensus models or evaluation protocols for assessing CT reconstruction quality currently exist. A promising initiative toward establishing benchmarking systems is the publication of real scan data acquired under multiple acquisition modes [41]. These datasets are employed to analyze and compare reconstruction algorithms under realistic low-dose, sparse-angle, and polychromatic scanning conditions [46]. A major strength of the work [46] lies in the availability of the published datasets and the accompanying open-source code.

An important study highlighting the significant lag of CT reconstruction research behind current best practices is [32]. Although the analysis was limited to image-domain LDCT denoising algorithms, it confirmed the presence of issues common across ML/DL research: the use of different datasets or dataset splits, unfair comparisons due to inconsistent hyperparameter choices, and inadequate quality metrics. The findings call into question the conclusions of a large number of prior studies, while the released code enables reproducibility and provides a foundation for fair comparison of new methods.

### 3.6. Conclusions on Publicly Available vCT Datasets

Analyzing Table 2, all publicly available volumetric vCT datasets are derived from clinical practice. For most published datasets (4 out of 5), authors use “best-quality reconstruction” as the GT for quantitative assessments, reflecting the pipeline for capturing real physical objects. These datasets are highly specialized, with each focusing on a few key modeling aspects, such as dose reduction or the presence/absence of metallic inclusions. When simulating a polychromatic source spectrum, the datasets typically provide sinograms after BHC, aligning with real-world medical systems. Moreover, even for publicly available datasets, access and downloading can be challenging (2 out of 5 datasets are publicly available).

In broad terms, a common significant issue in current vCT practices is the reliance on reconstructed medical CT images as the basis for generating data, which introduces artifacts into the simulated projections, calling into question the suitability of their reconstruction as GT. While many pipelines attempt to mitigate some of these artifacts, for example, through corrections like BHC, other methods often oversimplify polychromatic modeling into a monochromatic approximation, which fails to capture essential physical effects such as Compton scattering and beam hardening. More accurate approaches, such as true polychromatic modeling using spectral weighting of monochromatic projections or Monte Carlo simulations, could address these limitations. Another challenge lies in the limited accessibility of datasets, tools, and scripts, restricting reproducibility and innovation. Additionally, reprojecting volumes with artifacts requires careful selection of quality metrics and evaluation regions to ensure meaningful assessments. Transition to analytical GT derived from precise voxelized models could significantly enhance the quality and reliability of vCT datasets.

## 4. Flaws in Current Datasets and Benchmarks

A discussion of key considerations for establishing a unified training framework for reconstruction-domain LDCT methods is presented in [32]. While these insights apply to other tasks as well, many important aspects remain unaddressed. In the following, we examine the challenges related to datasets and evaluation protocols in a broader context—considering both general-purpose and task-specific approaches to CT reconstruction.

### 4.1. Limitations of Public Datasets and Benchmarking Protocols in CT Reconstruction

As emphasized above, for many tasks such as beam hardening correction, ring artifact suppression, scatter correction, and super-resolution, there are currently no publicly available CT datasets. For tasks that are supported by datasets, several challenges persist:

- There is no consensus on a representative dataset. Different studies use different datasets or subsets thereof. This issue is well illustrated for LDCT in [32], where the authors also propose a potential standardization strategy for reconstruction-domain LDCT methods. However, even this attempt does not resolve the issue of including projection- or dual-domain methods in comparative analyses.
- Different tasks rely on different base datasets, largely because there is no dataset with sufficient diversity in both imaging conditions and object types that could support cross-task evaluations (e.g., LDCT and MAR) or enable consistent benchmarking of various baseline CT reconstruction methods.

It is worth noting that vCT is actively used in the context of MAR. In contrast, the use of vCT for LDCT remains limited [65], likely due to the restricted availability of a single representative dataset. Moreover, the potential of vCT is largely overlooked by groups systematically working on benchmarking CT methods [32].

Even in studies where vCT is employed to simulate different dose levels [84], important aspects of the simulation process are often not described with sufficient clarity, and only the final reconstruction results are made available—without the corresponding projection data.

### 4.2. Constraints Imposed by Simplified vCT Models

Despite the advantages of vCT in terms of reproducibility and controllability, its utility is fundamentally constrained by the realism of the underlying simulation models. In particular, simplified assumptions about the source spectrum (e.g., purely monochromatic models), detector behavior, and system geometry may lead to discrepancies between simulated and real measurements [88]. Likewise, noise in vCT is often absent, Gaussian, or otherwise mismatched compared to the complex, energy-dependent, and spatially varying noise characteristics observed in xCT systems. The importance of realistic noise models is well highlighted in [89] and in [58], both showing that methods trained on simplified or mismatched noise distributions may perform well in simulations but degrade substantially on experimental data. Similarly, common artifacts such as beam hardening, scatter, or detector cross-talk are rarely modeled with sufficient fidelity, with significant implications for quantitative imaging accuracy [90]. Comparative studies also demonstrate that multi-energy CT simulations may diverge from real phantom measurements if oversimplified assumptions are applied to source spectra or material models [91]. As a result, reconstruction or artifact-correction methods trained or evaluated solely on vCT data may demonstrate good performance in simulations but fail to generalize to real-world xCT measurements.

These limitations underline the importance of developing more realistic simulation pipelines as a basis for vCT, combining polychromatic source models, observation-consistent noise generation, and artifact modeling, as well as validating vCT-trained methods against real xCT benchmarks whenever possible. For a more in-depth investigation of such modeling quality issues (e.g., the distinction between oversimplified and observation-consistent models), datasets that include both real raw measurements or synthetic sinograms (at all simulation stages) and corresponding digital phantoms are essential—precisely the type of resource advocated in this work.

### 4.3. Lack of Raw Data

In medical datasets, the de facto standard [65,66] involves providing sinograms after all conventional preprocessing steps, particularly those that include water correction (BHC). At the same time, the ability to access data in its minimally processed (or unprocessed, raw) form may be of interest to both developers of BHC methods and those who develop projection- or dual-domain task-oriented methods. Using vCT for dataset synthesis allows for obtaining raw data, with large data volume being the primary remaining limitation.

### 4.4. Using the Best Reconstruction as the GT

The need to use reconstructions under optimal acquisition conditions as GT is a characteristic of real-world measurements of patients or other objects with unknown internal structure. It is also important to note that using the best available reconstruction as GT inherently depends on the specific reconstruction method employed. As a result, learning-based approaches in such settings may unintentionally learn and reproduce artifacts specific to that method. The degree of influence exerted by the selected CT reconstruction method on the accuracy of reconstruction quality assessments and on the derived conclusions warrants additional analysis [46].

In contrast, vCT enables the use of analytically computed digital GT for a given reference energy of a monochromatic source; however, this approach has been adopted in only one publicly available dataset [65]. Furthermore, in an attempt to mitigate the influence of forward projection and reconstruction procedures on the GT, some challenges, such as the AAPM CT-MAR challenge [67], applied frequency boosting [92]. However, this led to a mismatch between the scanned object and the reconstructed results.

### 4.5. Data Verification

An excellent practice to assist users of datasets in preparing and testing reconstruction code is the provision of both sinograms and an example of the expected CT reconstruction for a test object [65]. This is particularly valuable when a digital twin of the test object exists, for instance, in the form of an analytical GT. Additionally, such data verification is useful for assessing the realism of the dataset and ensuring the absence of severe modeling artifacts that could render the dataset unsuitable for further utilization [5].

It is worth noting that the use of an analytical GT imposes higher requirements on the quality and automation of data validation. For example, inconsistencies in geometric parameters may go unnoticed if both the projections for the method under evaluation and those for the GT are generated using the same geometric configuration during simulation.

### 4.6. Implicit Specification of the Region of Interest and/or Its Inaccuracy

A typical CT reconstruction result generally represents a cylindrical volume, outside of which voxel values cannot be considered meaningful. Cropping to the inner volume of this cylinder [46] significantly reduces the usable region and is often infeasible, as it may exclude parts of the object of interest. A common compromise is to reduce the analyzed volume partially, leaving noisy artifacts outside the region of reconstruction. When quality assessment metrics are computed over the entire volume, such noise can distort the evaluation results; however, this issue is often overlooked in most studies.

To evaluate physically based characteristics, small regions are typically used—usually requiring prior knowledge of homogeneous areas or precise boundaries between distinct regions. Additionally, task-based evaluation metrics may be computed not over the full volume or slice, but within volumes of interest (VOIs) associated with relevant objects (e.g., lesions) or disease-related structures [32,65,84].

Therefore, we emphasize the importance of explicitly specifying the shape and size of VOIs in datasets, as comparisons between the reconstruction and GT outside these regions are meaningless. If the full volume cannot be used for evaluation, an additional, precisely defined evaluation protocol must be provided. This should include enforced masking of noisy regions or the use of metrics adapted for masked evaluation [5]. Accurate annotation of all regions used for metric computation should be explicit and constitute an integral part of both the dataset and the benchmarking procedure.

### 4.7. Vague Descriptions Without Publication of the Corresponding Quality Assessment Code

FR-IQA functions of general purpose, as implemented in popular libraries, are typically designed for images with intensity ranges of either [0.0,1.0] (floating-point) or [0,255] (integer). To enable proper evaluation, tomographic data must be normalized to the required range. This normalization may involve clipping values outside the specified bounds and applying linear rescaling. In medical imaging studies, normalization parameters are often defined in terms of a specific HU window selected for analysis.

It is important to note that many quality assessment functions involve additional hyperparameters, the default values of which may vary across different implementations.

In certain cases, such as when using a digital GT or in super-resolution tasks, the spatial resolution of the reconstruction results may differ from that of the GT. For instance, in [65], the reconstructed images are upsampled using linear interpolation to match the resolution of the digital phantom. Conversely, the digital GT can also be downscaled to the resolution of the reconstruction.

The quality assessment of CT images remains an important theoretical and practical issue to be addressed [75]. To ensure reproducibility, all decisions and rationales taken must be clearly and accurately documented, including any threshold values or constants used, as part of the dataset specification. In most cases, without the publication of the evaluation scripts, the risk of errors in reproduction significantly increases.

We next address the formalization of a data model for CT datasets, intended to facilitate the identification and documentation of domain-specific factors that compromise reproducibility.

## 5. Model

### 5.1. Basic Notation

Let us consider model representations of the physical volume in space. All coordinate systems (CSs) used hereafter are Cartesian, with points denoted as $\bar{p}=\left(p_{1},p_{2},p_{3}\right)\in R^{3}$. Let there be a global coordinate system (GCS), while all other CSs are defined as tuples of the form $c\overset{\mathrm{def}}{=}\langle \bar{0},{\bar{e}}_{1},{\bar{e}}_{2},{\bar{e}}_{3}\rangle$, where $\bar{0}$ denotes the origin of the CS *c* (and is represented by a point in GCS), $\bar{e_{i}},i\in I$, signify basis vectors of the CS *c* (defined in GCS), and $I\overset{\mathrm{def}}{=}\left\{1,2,3\right\}$ is the (ordinal) set of indices. The basis vectors of the CS are mutually orthogonal $\bar{e_{i}}\cdot \bar{e_{j}}=0$, are of equal unit length $|\bar{e_{i}}\left|=\right|\bar{e_{j}}|=1,\forall i\neq j$, and form a vector triad with the same orientation as the basis of the GCS, in particular, $\bar{e_{3}}=\bar{e_{1}}\times \bar{e_{2}}$.

A voxel volume with side lengths $\bar{n}\overset{\mathrm{def}}{=}\left(n_{1},n_{2},n_{3}\right)$ is defined as a tuple consisting of an image and a CS, $\langle I_{\bar{n}}^{3},c\rangle$, where the image is a mapping $I_{\bar{n}}^{3}:Z_{\bar{n}}^{3}\to T$. The domain $Z_{\bar{n}}^{3}\overset{\mathrm{def}}{=}\prod_{i=1}^{3}\left\{0,1,\ldots ,n_{i}-1\right\}\subset R^{3}$ is called the raster, with $n_{i}\in N$, and *T* denotes the voxel value type, which includes a special value Ø.

A tuple $\langle \bar{z},a\rangle$, where $\bar{z}\in Z_{\bar{n}}^{3}$, a∈T, is called a voxel. The vector $\bar{z}$ is referred to as the voxel position, its elements as the voxel coordinates, and *a* as the voxel value, with the special value Ø indicating the absence of information. In the coordinate system of the voxel volume *c*, a voxel is interpreted as a cubic volume whose center is specified by the voxel coordinates, and whose edge length coincides with the length of a basis vector.

Hereafter, without loss of generality, we consider rigid objects, for which the object’s own coordinate system (OCS) is rigidly attached to the object. We use the notation $\bar{p}\in \left[c\right]$ or ${\bar{p}}_{\left[c\right]}$ to indicate that the coordinates of point $\bar{p}$ are expressed in the coordinate system *c*. For two given coordinate systems $c_{1}$ and $c_{2}$, the transformation that converts the coordinates of points from $c_{1}$ to $c_{2}$ is denoted by $g_{\left[c_{2},c_{1}\right]}$, i.e., ${\bar{p}}_{\left[c_{2}\right]}=g_{\left[c_{2},c_{1}\right]}{\bar{p}}_{\left[c_{1}\right]}$. Moreover, a chain of successive coordinate transformations can be simplified as $g_{\left[c_{3},c_{2}\right]}g_{\left[c_{2},c_{1}\right]}=g_{\left[c_{3},c_{1}\right]}$.

### 5.2. Digital (Object) Model

The spatial distribution of the physical object is described by *n* materials, specified by the identifier vector $\bar{m}\overset{\mathrm{def}}{=}\left(m_{1},\ldots ,m_{n}\right)$. We will use the following types, ordered by decreasing potentially achievable accuracy of representing a digital model of the object:

- $V_{\left[c,\bar{m}\right]}$—vector model in the coordinate system *c*;
- $W_{\left[c,\bar{m}\right]}$—material weight mask in the coordinate system *c*;
- $S_{\left[c,\bar{m}\right]}$—segmentation mask in the coordinate system *c*;
- $R_{\left[c,e\right]}$—reconstruction (LAC values) in the coordinate system *c* at a given reference energy *e*;
- $H_{\left[c\right]}$—reconstruction in HU units in the coordinate system *c*;
- $I_{\left[c,w\right]}$—reconstruction in the coordinate system *c* with a given HU-window w.

**Figure 1:**
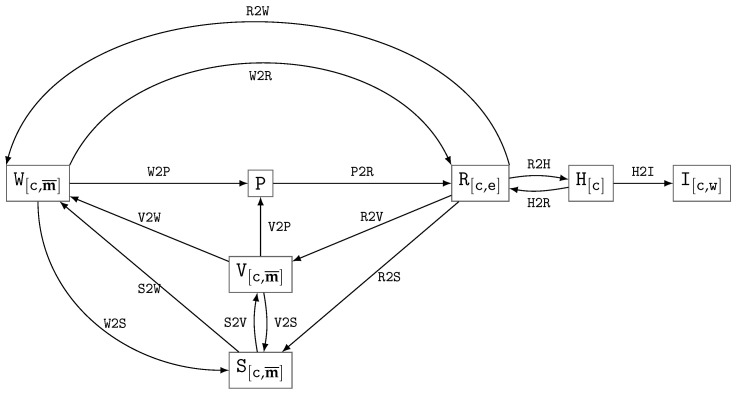
Diagram of the notation for the main data types (representations) and the transformations between them in the digital object model in vCT; the arcs represent the transformations, while boxes stand for data types.

The explicit indication of the CS, material vector, and reference energy may hereafter be omitted when not required by the context. From the perspective of CT tasks, typical transformations between the described types and sinograms P are shown in Figure 1; the identifiers of the transformations are indicated on the edges and are examined in more detail in Table 3 and subsequently explained in the text.

The vector model $v_{\left[c\right]}\in V_{\left[c\right]}$ describes the material distribution in the volume by means of analytical functions. Specific methods of representation and manipulation are provided by CAD technologies: sets of primitives and operation trees, spline-based or polygonal/triangulated boundary surfaces. Voxels of the material weight mask $w_{\left[c,\bar{m}\right]}\in W_{\left[c,\bar{m}\right]}$ contain vectors of the form $\bar{w}\overset{\mathrm{def}}{=}\left(w_{1},w_{2},\ldots ,w_{n}\right)$, where $w_{i}\in \left[0.0,1.0\right]$ is the weight of material *i*, subject to the constraint $\sum_{i=1}^{n}w_{i}\leq 1.0$. Voxels of the segmentation mask $s_{\left[c,\bar{m}\right]}\in S_{\left[c,\bar{m}\right]}$ store the index of the assigned material class. And the reconstruction voxels $r_{\left[c,e\right]}\in R_{\left[c,e\right]}$ carry the LAC values at a given energy *e*, or their corresponding HU values $h_{\left[c,e\right]}\in H_{\left[c\right]}$.

In medical applications, both for visualization and analysis, windowing of HU values is employed, defined by the HU-window $w\overset{\mathrm{def}}{=}\langle l,\omega \rangle$, where *l* is the level and ω is its width. This HU-windowing is related to the visualization $i_{\left[c,w\right]}\in I_{\left[c,w\right]}$. Without loss of generality, the representations H and I will henceforth be omitted in cases where their transformations are analogous to those of R; further details on their use can be found in Section 5.4.

A sinogram p∈P represents a set of projection images for a given set of acquisition angles, obtained as the result of scanning either the entire object or only a portion of it, if the object does not fully fit within the field of view (FOV). In the general case, the sinogram is assumed to contain reconstruction-ready values (logarithmized and normalized). The notation $p^{raw}$ will be utilized to denote the raw projection measurement results, supplemented with the air scan, dark field scan, and the exact description/code of the procedure used to generate the prepared reconstruction-ready sinogram.

A digital phantom is defined as a description of the scanned region used by a CT simulator (vCT scanner) for sinogram calculation. Depending on the simulation method, the phantom may be voxel-based w∈W, e.g., [73], or vector-based v∈V, e.g., [93].

### 5.3. Data Transformations

Data transformations between different types may occur with varying degrees of reproducibility, which can be classified into one of the following three levels:

- det—weakly variable (the transformation algorithm is unambiguous, and when fixed parameters are used, a high degree of reproducibility at level L2 is achieved);
- var—highly variable (the transformation may be performed in different ways, and algorithms of varying degrees of accuracy exist; to achieve reproducibility beyond level L1, a precise description is required);
- rnd—randomized (the algorithms are specifically designed to model stochastic processes, in particular physical phenomena such as absorption, scattering, etc.; reproducibility requires not only detailed descriptions/code but also the ability to control the random seed).

The notation, variability level, and a brief description of the transformations are presented in Table 3.

The segmentation mask $f_{\left[c\right]}^{VOI}\in S$ is specific to the scanned object and delineates regions of interest (ROIs) within that object. In medical applications, such regions may correspond to lesions, tumors, or similar structures, as well as to areas of homogeneous material or tissue. The binary mask $f_{\left[c\right]}^{FOV}$ represents the field of view (FOV), which is determined by the scanning geometry and does not depend on the scanned object; in the general case, it has a cylindrical shape. Without loss of generality, we assume the operation $f_{\left[c\right]}^{FOV}=\mathrm{FOV}\left(r_{\left[c,e\right]}\right)$ to be well defined, based on the values of non-informative pixels stored in the DICOM (Digital Imaging and Communications in Medicine) format, and also allowing for alternative methods of encoding this information within tomographic reconstruction pipelines.

### 5.4. Specifics of Using HU Units and Windowing

The optical density of materials in the X-ray range is significantly influenced by photon energy, meaning that HU values are not conserved when the reference energy is changed. For example, Table 4 compares the HU values of several materials simulated using XCIST for reference energies $e_{ref}=80\;\mathrm{kEv}$ and $e_{ref}=120\;\mathrm{kEv}$.

As a result, the same object (e.g., a specific anatomical region of a patient) will have varying voxel values depending on the photon energy used during scanning. This effect is particularly pronounced for denser materials.

It is important to note that in medical imaging, CT data processing often involves a procedure called windowing. Windowing defines the HU range by selecting reconstruction regions nominally corresponding to HU values 0 and 1, with the brightness of other regions linearly scaled between 0 and 1. When calculating image quality assessment metrics, it is crucial to distinguish whether the data being processed is pre- or post-window normalized. HU values can be derived either from window-normalized data or from the original data, and this distinction can significantly impact the results of quality assessments.

CT data processing frequently involves multiple recalculations with specific coefficients (e.g., the HU value for air). It is essential to explicitly and precisely define these coefficients during reconstruction and quality assessment procedures (e.g., in DICOM metadata). To ensure accurate quality assessments, the data processing workflow must be detailed. For reproducibility in quality evaluation, either the processing code or specific data annotations (e.g., in DICOM) should be provided.

The inverse transformation H2R is implemented by inverting Expressions (1) or (2). In practice, the need for such a transformation arises at the stage of preparing a digital phantom from reconstructions in HU, for which it is recommended [59] to perform dequantization with normalization of CS and the addition of low-amplitude noise.

### 5.5. Types of Functions for Assessing Reconstruction Quality

Within the proposed model, depending on the data used to compute quality assessment functions, we identify five types of such functions (Table 5), for which the following notation will be used.

For quality evaluation, the input data are brought into a common CS. It should be noted that the computations must take into account the regions of relevant voxels, for example, $f_{\left[c\right]}^{Q^{FR}}=\mathrm{FOV}\left(r_{\left[c,e\right]}^{GT}\right)\cap \mathrm{FOV}\left(r_{\left[c,e\right]}\right)$ or $f_{\left[c\right]}^{Q^{TF}}=\mathrm{FOV}\left(r_{\left[c,e\right]}^{GT}\right)\cap \mathrm{FOV}\left(r_{\left[c,e\right]}\right)\cap f_{\left[c\right]}^{VOI_{GT}}$. The choice of averaging strategy across images and/or regions of interest is also critical for reproducibility.

### 5.6. Model for the Assessment of xCT Reconstruction Quality

**Figure 2:**
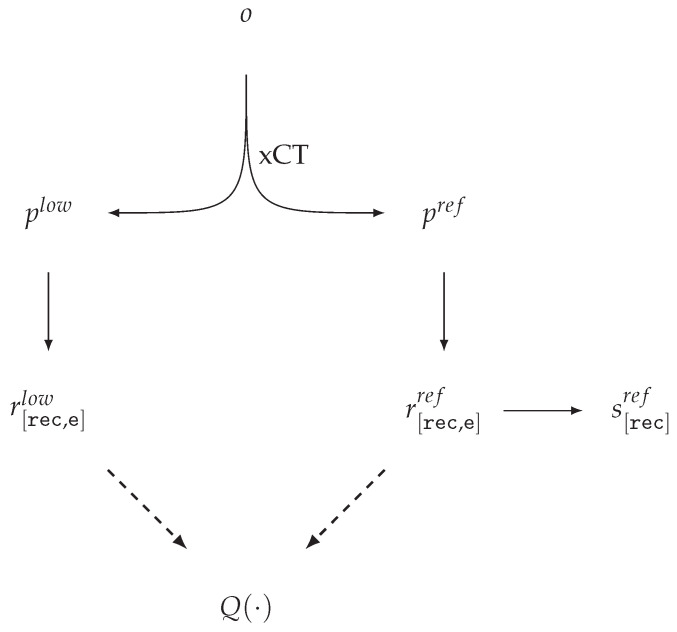
Scheme of the xCT experiment with multiple measurements for a single object, for the 2DeteCT dataset, according to references [41,46]. In this and the following figures, black solid arrows indicate data transformations within the main pipeline, while black dashed arrows represent the use of data for quality assessment.

In the terminology introduced above, we outline the workflow for dataset and benchmark construction [41,46] in which, for a physical object *o* with a given position, xCT acquisitions yield high- and low-quality sinograms $p^{ref}$ and $p^{low}$, with the corresponding reconstructions $r_{\left[\mathrm{rec},e\right]}^{ref}$ and $r_{\left[\mathrm{rec},e\right]}^{low}$ (Figure 2).

For real measurements, a challenging technical task is to maintain a consistent CS across all projection sets and their corresponding reconstructions. In reference [41], a unified coordinate system is ensured at the level of the acquisition protocol and hardware. As a result, the corresponding projections $p^{low},p^{ref}$ and reconstructions $r_{\left[\mathrm{rec},e\right]}^{low},r_{\left[\mathrm{rec},e\right]}^{ref}$ are geometrically aligned in the CS rec, while the mask $f_{\left[\mathrm{rec}\right]}^{FOV}$ is fixed at the dataset specification level. For $r_{\left[\mathrm{rec},e\right]}^{ref}$, the authors provide a segmentation mask $s_{\left[\mathrm{rec}\right]}^{ref}$, which, however, is not used in quality evaluation. Numerical quality assessments [46] are performed using functions of type $Q^{FR}$, which introduces dependence on the selected reference acquisition settings and reconstruction algorithm. This dependence is explicitly highlighted by the authors themselves as a limitation.

### 5.7. A Model for Assessing CT Reconstruction When Sinogram Modification Is Used

**Figure 3:**
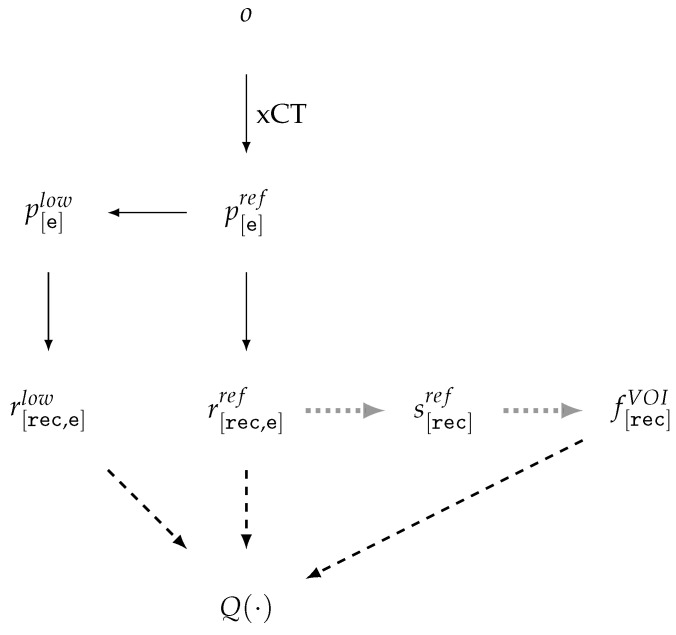
Scheme of the dataset preparation and benchmarking with data generation via modification (degradation) of real sinograms. In this and the following figures, gray dotted arrows denote additional potential data transformations.

For data generation [46,59,68] in low-dose, sparse-angle, limited-angle, and any-time CT tasks, a lower-quality sinogram $p_{\left[e\right]}^{low}$ is generated from a high-quality reference sinogram $p_{\left[e\right]}^{ref}$, followed by reconstruction and comparison of the results (Figure 3).

Quality reduction is achieved by subsampling projections DSS, RSS and/or adding noise to the projections NOI. Within this scheme, both the sinograms themselves and corresponding reconstructions, $r_{\left[\mathrm{rec},e\right]}^{ref}$ and $r_{\left[\mathrm{rec},e\right]}^{low}$, are geometrically consistent. Denoising DEN constitutes an integral part of the reconstruction process, although it is often treated as a separate step—either as sinogram preprocessing or as postprocessing of the reconstructions. The segmentation mask $s_{\left[\mathrm{rec}\right]}^{ref}$, generated during the preparation stage, is regarded as part of the reference, along with $r_{\left[\mathrm{rec},e\right]}^{ref}$, and subsequently, the quality of reconstruction algorithms is evaluated using functions of types $Q^{FR},Q^{TF}$.

### 5.8. A Model for Assessing CT Reconstruction in the MAR Task

**Figure 4:**
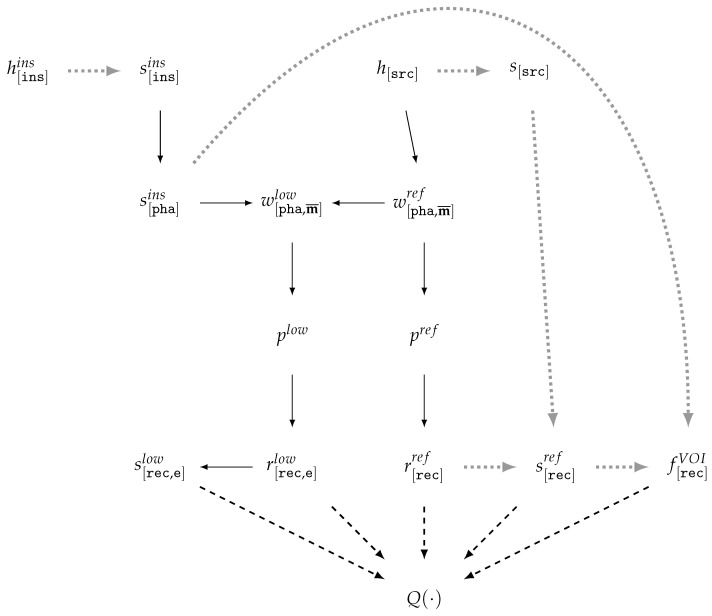
Experimental setup for MAR data generation in vCT.

According to the analysis of prior studies [61,66,96], in the MAR task, the scheme for CT data generation and reconstruction quality evaluation (Figure 4) is based on modeling the CT scanning process of two phantom versions that differ by the presence of a highly attenuating inclusion.

In the data preparation stage (Figure 4), to enhance the realism of vCT, real patient tomographic reconstructions $h_{\left[\mathrm{src}\right]}$ are taken as the source, along with reconstructions $h_{\left[\mathrm{ins}\right]}^{ins}$ with binary masks $s_{\left[\mathrm{ins}\right]}^{ins}$ of highly attenuating inclusions. Simple methods for generating the material weighting mask $w_{\left[\mathrm{pha},\bar{m}\right]}^{ref}$ from $h_{\left[\mathrm{src}\right]}$ are based on the soft threshold-based weighting method [97] for a selected reference energy (see Section 5.4). More accurate and complex material masks can be obtained from neural network segmentation results, for example, as in [98]. The inclusion mask $s_{\left[\mathrm{pha}\right]}^{ins}$ is positioned within the phantom volume and combined with the phantom without inclusion $w_{\left[\mathrm{pha},\bar{m}\right]}^{ref}$ to generate the phantom with inclusion $w_{\left[\mathrm{pha},\bar{m}\right]}^{low}$. The preparation of the volume-of-interest mask $f_{\left[\mathrm{rec}\right]}^{VOI}$ can be carried out, taking into account the segmentation masks $s_{\left[\mathrm{src}\right]}$ and/or $s_{\left[\mathrm{rec}\right]}^{ref}$, with the use of the inclusion position $s_{\left[\mathrm{pha}\right]}^{ins}$. In published works, the generation scheme for $w_{\left[\mathrm{pha},\bar{m}\right]}^{low}$ and $f_{\left[\mathrm{rec}\right]}^{VOI}$ is insufficiently detailed to guarantee reproducibility. The sinograms $p^{low},p^{ref}$ are generated using a vCT simulator modeling the polychromatic source spectrum. The sinograms may be produced either with BHC [64,67] or without BHC [61]. The reconstruction results $r_{\left[\mathrm{rec},e\right]}^{low}$, $r_{\left[\mathrm{rec},e\right]}^{ref}$ obtained by the reference and investigated methods are geometrically consistent. These reconstructions, together with $f_{\left[\mathrm{rec}\right]}^{VOI}$, are used for quality evaluation by means of $Q^{FR},Q^{TF},Q^{TM}$.

### 5.9. A Model for Assessing CT Reconstruction Using Vector Models and vCT (TrueCT)

**Figure 5:**
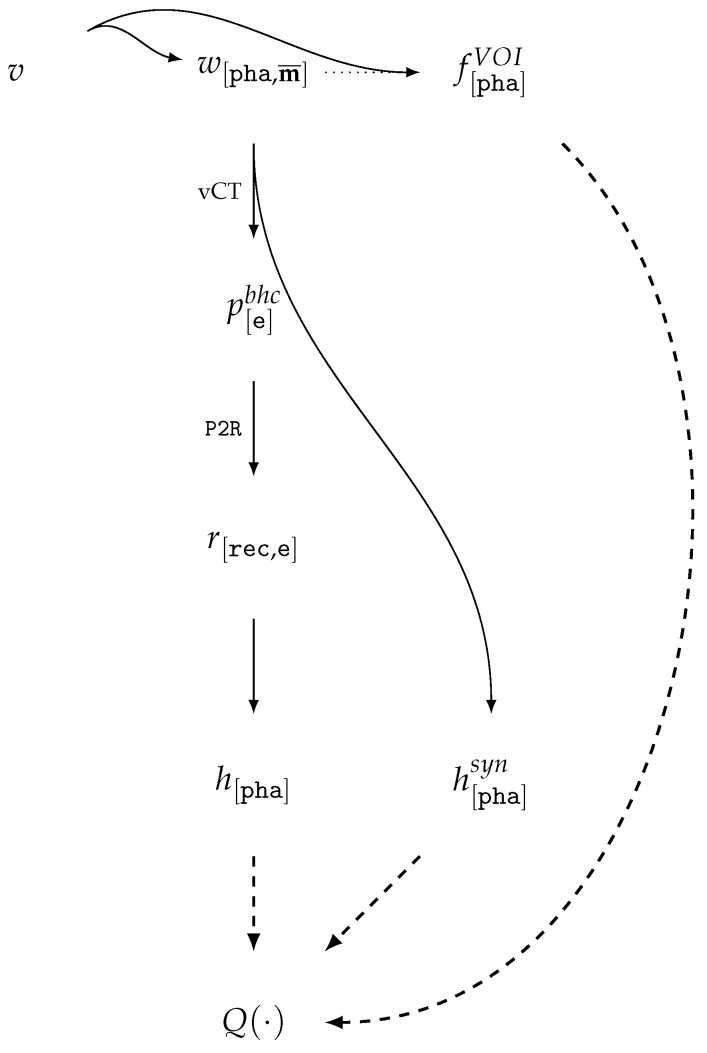
TrueCT modeling scheme.

Let us consider the vCT scheme presented in [65] (Figure 5), where the vector model *v* is voxelized within a given volume to obtain a digital phantom $w_{\left[\mathrm{pha},\bar{m}\right]}$ and a volume-of-interest mask $f_{\left[\mathrm{pha}\right]}^{VOI}$. In this setup, the reference energy *e* of a monoenergetic source, consistent with W2P, P2R, together with the material dictionary, is used to compute the analytical reconstruction result in HU units taken as the ground truth, $h_{\left[\mathrm{pha}\right]}^{syn}=R2H\left(W2R_{\left[e\right]}\left(w_{\left[\mathrm{pha},\bar{m}\right]}\right)\right)$.

The sinogram $p_{\left[e\right]}^{bhc}={\mathrm{BHC}}_{\left[e\right]}\left(W2P\left(w_{\left[\mathrm{pha},\bar{m}\right]}\right)\right)$ is generated by a virtual scanner with source spectrum modeling and water correction, i.e., it contains normalized values after BHC. The reconstruction results $r_{\left[\mathrm{rec},e\right]}=P2R\left(p_{\left[e\right]}^{bhc}\right)$ are mapped to the phantom coordinate system (by upscaling to the voxel size of the corresponding human models using linear interpolation) and converted into HU units $h_{\left[\mathrm{pha}\right]}=R2H\left(\mathrm{RES}\left(r_{\left[\mathrm{rec},e\right]}\right)\right)$ at the effective acquisition energy. The quality evaluation functions $Q^{FR},Q^{TF},Q^{TM}$ are computed for reconstructions in HU units expressed in the phantom coordinate system and with the use of the volume-of-interest masks $f_{\left[\mathrm{pha}\right]}^{VOI}$. It should be noted that the use of the phantom coordinate system for evaluation [65] is a design choice of the cited study’s authors and is neither justified nor validated.

## 6. Extended Frameworks for Data Preparation and Benchmarking

Section 2.1 outlines a broad range of specific task formulations that are commonly treated as CT quality improvement tasks, yet, as demonstrated in Section 3, no open datasets or benchmarks are available for them. The list of data-deficient tasks can be further extended by:

- selection of optimal acquisition modes and parameters, including object orientation, projection angles, source spectrum, exposure, etc.;
- evaluation of the robustness of learning-based reconstruction methods to input variability, including robustness to adversarial attacks [99].

Along with this, in most cases, CT reconstruction is not the final output of the pipeline. From the users’ perspective, the ultimate goal is not merely the quality of the reconstruction itself, but rather the quality of results and performance in one or more downstream tasks, as listed in Table 6.

Expanding the number of tasks covered by datasets requires extending the data preparation workflows for benchmarking. The proposed variants of the workflows will be examined in detail for xCT (Section 6.1) and vCT (Section 6.2).

### 6.1. Extended Model for CT Data Evaluation in xCT Scanning

**Figure 6:**
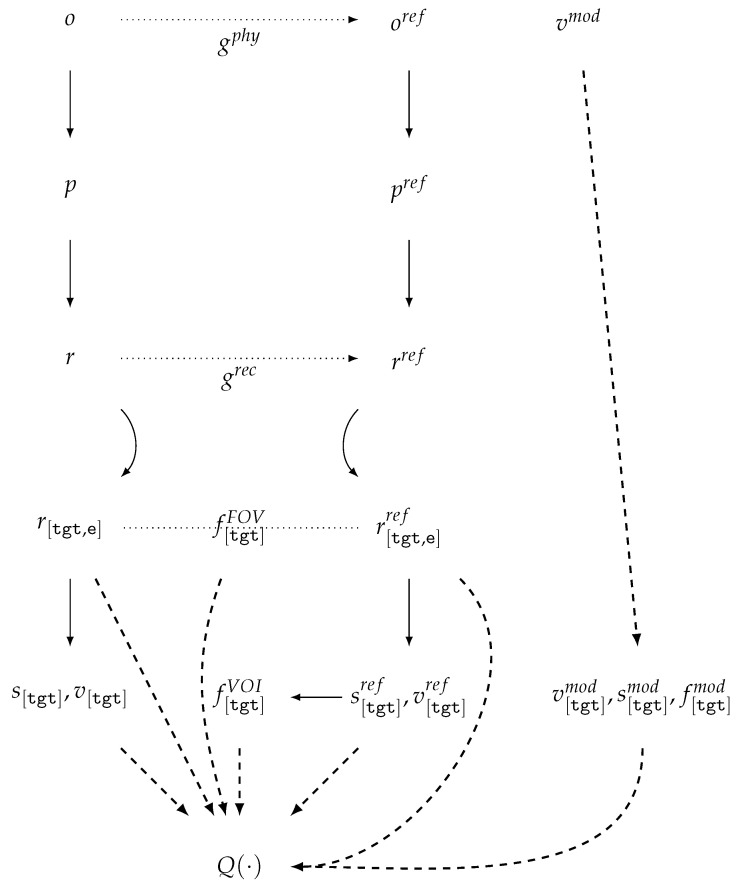
Generalized workflow for extended xCT experimentation.

The proposed xCT data preparation workflow is illustrated in Figure 6, where, in the general case, a rigid (non-deformable) object is scanned in a pair of arbitrary orientations *o* and $o^{ref}$.

Such a workflow makes it possible to evaluate the impact of object orientation during CT scanning on reconstruction quality, which is particularly relevant for indCT. In this case, there is no natural unified coordinate system CS for the reconstructions; therefore, the target CS tgt is chosen for reasons of convenience (a task-dependent CS). This CS is aligned with the intended downstream use of the reconstructions and, in the general case, does not coincide with either the phantom CS or the reconstruction CS. In medical applications, an example of this approach is the normalization of reconstruction spatial resolution for the calculation of radiomics features [36,38].

When the experimental setup allows, the parameters $g^{phy}$ describing changes in the geometric position of the object in space are measured via instrumental means. Under general conditions, no restrictions are imposed on either the measurement scheme or the method of measurement. Even when a single measurement system is employed, the sinograms $p,p^{ref}$ and their corresponding reconstructions $r,r^{ref}$ remain geometrically independent. For quality assessment, the workflow must include an image registration stage, which yields an estimate of the geometric alignment parameters $g^{rec}$ for co-registration of *r* and $r^{ref}$. Such an estimation may be performed either with the aid of $g^{phy}$ or independently of it. In general, all reconstructions are transformed into a common target CS tgt and reference energy *e*, resulting in $r_{\left[\mathrm{tgt}\right]},r_{\left[\mathrm{tgt}\right]}^{ref}$. The region of the common field of view is defined as $f_{\left[\mathrm{tgt}\right]}^{FOV}=\mathrm{FOV}\left(r_{\left[\mathrm{tgt}\right]}\right)\cap \mathrm{FOV}\left(r_{\left[\mathrm{tgt}\right]}^{ref}\right)$. During the data preparation stage, the segmentation map $s_{\left[\mathrm{tgt}\right]}^{ref}$ together with the vector models $v_{\left[\mathrm{tgt}\right]}^{ref}$ of the object are utilized to derive precise annotations of the regions of interest $f_{\left[\mathrm{tgt}\right]}^{VOI}$. In contrast to Section 5.6, we propose extending the evaluation beyond $Q^{FR}$ to include task-specific metrics computed within the regions of interest $Q^{TF}$, as well as system-level quality measures $Q^{TD}$.

If a prior vector model of the object *v* is available, denoted by $v_{\left[\mathrm{tgt}\right]}$, it can be aligned with the reconstruction results $r_{\left[\mathrm{tgt}\right]}^{ref}$. From this alignment, segmentation masks $s_{\left[\mathrm{tgt}\right]}^{mod}$, VOIs $f_{\left[\mathrm{tgt}\right]}^{mod}$, and the aligned vector model itself $v_{\left[\mathrm{tgt}\right]}^{mod}$ can be derived and subsequently used for computing the task-specific model-based quality assessment functions $Q^{TM}$. This type of approach is applied for automatic or semi-automatic quality control of medical CT reconstructions using physical phantoms [100].

### 6.2. Extended Model for CT Data Evaluation in vCT Scanning

**Figure 7:**
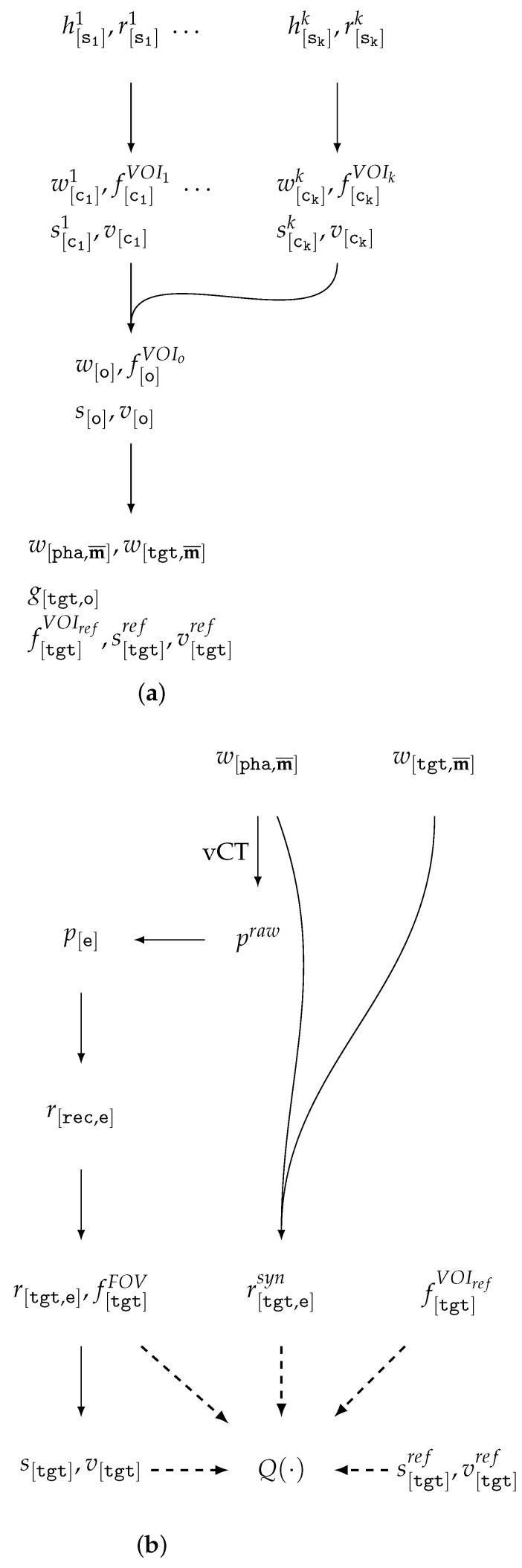
vCT workflow for benchmarking: (**a**) data preparation, (**b**) quality assessment.

Let us consider the data preparation and benchmarking workflow based on voxel-based data, since for vector models, the workflow is simpler (due to voxelization into predefined CSs). The preparation of vCT data can be conveniently divided into two stages: the construction of the digital phantom (see Figure 7a) and the actual vCT simulation (see Figure 7b).

The initial stage of data preparation is similar to Section 5.8: for each original reconstruction $h_{\left[s_{i}\right]}^{i}$ or $r_{\left[s_{i}\right]}^{i}$, spatial normalization may be performed (if necessary) to enforce axis-wise uniformity of the CS, along with dequantization achieved through CS adjustment and the addition of low-amplitude noise to the values. Subsequently, a material decomposition $w_{\left[c_{i}\right]}$ and a mask of regions of interest $f_{\left[c_{i}\right]}^{VOI_{i}}$ are generated in the normalized coordinate system $c_{i}$. At this stage, reference segmentation maps $s_{\left[c_{i}\right]}^{i}$ and vector models $v_{\left[c_{i}\right]}^{i}$ may also be produced.

We propose to consider the next stage in a more general form as the combination of multiple objects to generate a digital twin of the object $\left(w_{\left[o\right]},f_{\left[o\right]}^{VOI_{o}},s_{\left[o\right]},v_{\left[o\right]}\right)$ in the coordinate system *o*. This approach allows for modeling of not only typical medCT insertions, such as “metallic inclusions” or tumors, but also of scenarios closer to indCT, for example, placing the object within a shell or container.

For the vCT scanning itself, a phantom $w_{\left[\mathrm{pha},\bar{m}\right]}$ is generated as a WMM $w_{\left[\mathrm{pha},\bar{m}\right]}$ within the scanning region in the CS pha, representing the object or its region in a given orientation. The result of the combination may include $w_{\left[\mathrm{tgt},\bar{m}\right]}$, as well as $g_{\left[\mathrm{tgt},o\right]}$, which is then used for the automatic derivation of the reference description $f_{\left[\mathrm{tgt}\right]}^{VOI_{ref}},s_{\left[\mathrm{tgt}\right]}^{ref},v_{\left[\mathrm{tgt}\right]}^{ref}$.

The target coordinate system tgt is defined as common to all phantoms whose reconstruction results are intended for subsequent comparison. If the CS tgt is not known at the phantom generation stage, then $f_{\left[\mathrm{pha}\right]}^{VOI_{ref}},s_{\left[\mathrm{pha}\right]}^{ref},v_{\left[\mathrm{pha}\right]}^{ref}$ may become the artifacts stored as part of the dataset.

When simulating the raw sinogram $p^{raw}=W2P_{\left[\bar{e}\right]}\left(w_{\left[\mathrm{pha},\bar{m}\right]}\right)$ for a polychromatic source spectrum $\bar{e}$, a reference energy $e=e_{ref}$ is fixed, at which the analytical reconstruction $r_{\left[\mathrm{tgt},e\right]}^{syn}=g_{\left[\mathrm{tgt},o\right]}\left(W2R_{\left[e\right]}\left(w_{\left[o,\bar{m}\right]}\right)\right)$ is computed. To improve reproducibility, $r_{\left[o,e\right]}^{syn}$ or $r_{\left[\mathrm{pha},e\right]}^{syn}$ may be considered as part of the dataset.

The computation of the sequence of possible corrections and transformations of the sinogram between the raw version $p^{raw}$ and the reconstruction-ready version $p_{\left[e\right]}$ requires a certain baseline as part of the benchmark. Moreover, the sinograms at each stage should be considered part of the dataset to enhance reproducibility.

Since $g_{\left[\mathrm{tgt},o\right]}$ and $g_{\left[o,\mathrm{rec}\right]}$ are determined by the generation procedure and the geometric parameters of the vCT, the transformation parameters $g_{\left[\mathrm{tgt},\mathrm{rec}\right]}=g_{\left[\mathrm{tgt},o\right]}g_{\left[o,\mathrm{rec}\right]}$ for $r_{\left[\mathrm{tgt},e\right]}={\mathrm{RES}}_{\left[\mathrm{tgt},\mathrm{rec}\right]}\left(r_{\left[\mathrm{rec},e\right]}\right)$ can be readily computed. It is worth noting that the explicit preservation of geometric transformation parameters in the dataset for different orientations of the same phantom object enables the evaluation of registration, alignment, stitching, etc.

After constructing $s_{\left[\mathrm{tgt}\right]},v_{\left[\mathrm{tgt}\right]}$, all types of quality assessment functions (Section 5.5) can be applied to evaluate the reconstruction results, utilizing $f_{\left[\mathrm{tgt}\right]}^{VOI_{ref}}$ and the GT in the form of $r_{\left[\mathrm{tgt},e\right]}^{syn},s_{\left[\mathrm{tgt}\right]}^{ref},v_{\left[\mathrm{tgt}\right]}^{ref}$.

### 6.3. Proposals for Practices in CT Dataset and Benchmark Creation Conducive to Reproducibility

At the current stage, one of the limiting factors in the development of CT reconstruction methods is the lack of publicly available volumetric datasets with high variability for training and benchmarking, which would allow for objective and reliable comparison of different methods. A primary source of such data could be highly realistic virtual vCT simulations. Nevertheless, existing data preparation practices typically follow straightforward pipelines for scanning real-world physical objects and do not fully exploit the potential offered by vCT.

Three out of the four evaluation models (Section 5.6, Section 5.7 and Section 5.8) exploit their inherent consistency of CSs of the resulting reconstructions. Several authors have noted the substantial dependence of these models on the choice of the underlying reconstruction method. However, even for image-domain artifact correction approaches, no studies have investigated the hypothesis that the ranking of method quality is independent of the selected baseline reconstruction algorithm.

The essential components and conditions for reproducible CT data modeling and quality assessment are summarized in Table 7, serving as a practical checklist for data preparation and evaluation. The statements listed in this table may be used to construct a checklist, with each item rated according to one of three options: yes, no, or not applicable.

In medCT, representativeness is often defined in terms of the balance and coverage of patient cohorts across various attributes commonly used in medical studies (e.g., sex, age, height, weight, diagnosis, ethnicity, etc.). Both the data preparation models and the datasets under consideration are tailored to medCT, thereby considerably restricting their utility for indCT tasks. IndCT requires the development of models and methods to describe the variability of objects, acquisition protocols, and reconstruction outcomes, enabling the creation of balanced samples and the accurate tracking of benchmark applicability limits. This task is further complicated by the fact that visually similar reconstruction artifacts may arise from different underlying data characteristics.

## 7. Future Works

### 7.1. Enhancing Reproducibility

Raising awareness of reproducibility issues can be achieved through information dissemination, the development of informational models, checklists, and publication policies.

A critical step in advancing CT reconstruction methods may be the adoption of practices established in the ML/DL community, such as replication or independent reproduction of published results using publicly available datasets and benchmarks. Refinement of data generation and processing models in xCT/vCT pipelines, along with the identification of key drivers for enhancing reproducibility, is essential. Compliance with the components and conditions outlined in Section 6.3 in data preparation workflows and benchmark utilization is a key component of this effort.

Another key avenue for improving reproducibility is narrowing the gap between xCT and vCT results. Some studies addressing this issue have already been conducted, with results available to the research community [73,101]. Nonetheless, there is currently no established practice for publishing raw measurement data, which hampers the creation of more accurate models of CT systems and their individual components, including realistic, observation-consistent modeling of component degradation.

A major contribution to reproducibility could come from the development of new pairs of physical objects and their digital twins, for which coordinated xCT and vCT measurements are performed. Publishing these results across a wide range of acquisition conditions, imaging modes, and system components would enable benchmarking based on these datasets.

Accumulation of such coordinated datasets would allow for the verification of simplified projection and reconstruction modeling methods, which are often proposed to overcome the inherent computational challenges associated with increasing the realism of vCT simulations.

At the same time, one of the most practical barriers to reproducibility remains the substantial computational cost of high-fidelity vCT modeling. The simulation of realistic physics, spectral characteristics, and system imperfections often demands high-performance computing resources, which limits the accessibility of these methods to well-funded laboratories. Addressing this obstacle requires both the development of more efficient surrogate models and community practices for sharing pre-computed reference datasets to reduce redundant computational efforts.

A key indicator of success in this direction would be improved reproducibility of methods trained on vCT data when applied to real xCT measurements.

### 7.2. Development of Evaluation Methods

The use of analytical GT is currently underrepresented in studies. Existing quality assessment metrics and loss functions in ML/DL methods may require additional adaptation, as reproducing such reference results is, on the one hand, independent of the choice of the underlying reconstruction method, but on the other hand, fundamentally unattainable. The best achievable reconstruction outcome typically lies somewhere between the analytical GT and the reconstruction under optimal conditions.

To standardize evaluation methods and metrics, a more systematic approach is required—one that considers the evaluation task more comprehensively, taking into account potential downstream tasks and formally defining the limits of variability in objects, acquisition protocols, and sinograms.

Given the wide range of applied tasks addressed using CT reconstruction, a single, universally correct measure of quality is not feasible. Therefore, the future lies in the development of a standardized set of baseline evaluation metrics and task-specific ranking methods that aggregate assessment results based on such metrics.

Attention must also be given to the reproducibility of the quality assessment procedures themselves, which should be reasonably tolerant to the inherent variability of CT reconstruction results. In this context, the creation of open-access datasets and benchmarks for the verification of evaluation metrics and ranking methods becomes momentous.

### 7.3. Creating New Open Multitask and Multidomain CT Datasets

A critical component of the initiatives outlined above is the creation and publication of datasets that incorporate the most comprehensive data possible. Such datasets should include the original digital models and phantoms, raw measurement data, intermediate projection-processing results, reconstructed volumes, as well as detailed records of geometric configurations and parameters for both xCT acquisition and vCT simulations.

To overcome domain boundaries, these datasets must also contain a series of measurements of the same object acquired under a wide range of positions, conditions, and scanning regimes. The systematic inclusion of such series—particularly for calibration objects—would not only facilitate debugging of CT reconstruction pipelines but also expand the repertoire of available evaluation functions.

As the scope of accessible datasets grows, the issue of selecting and analyzing representative subsets will become increasingly critical. On the one hand, careful curation is required to ensure high representativeness; on the other, it is equally important to define clearly the boundaries of dataset applicability. Addressing these challenges calls for the development of formal ontological models for describing both tomographic measurements and reconstructions. The ability to capture variability in this way will, in the future, enable the construction of targeted subsets with predefined distributions of geometric configurations, material compositions, scanning parameters, equipment characteristics, and similar factors.

Collectively, these efforts should lay the groundwork for establishing shared benchmarking protocols that support objective, reproducible, and informative comparisons of reconstruction methods. Such protocols will enable the systematic tracking of scientific and technological progress through open evaluation tools while also providing practitioners with a sound basis for selecting the most effective methods and practices for their specific application needs.

Nevertheless, ethical and legal aspects must also be considered, particularly in the context of publishing medical CT data. Patient privacy, consent, and data protection regulations (such as GDPR and HIPAA) place strict limits on data sharing. These issues complicate the creation of openly accessible medical benchmarks and require solutions such as anonymization, federated learning frameworks, or the use of hybrid datasets combining real and synthetic scans. Without explicit attention to these concerns, efforts toward open and reproducible benchmarking risk encountering significant barriers to adoption.

## 8. Conclusions

In this work, we reviewed the current state of benchmarking practices in CT. The situation remains unsatisfactory: publicly available datasets are scarce, and those that do exist are often incomplete in their description or tailored to a narrow range of tasks while also suffering from methodological shortcomings. Methods commonly used by CT researchers—including quality assessment techniques—are either not published at all or disseminated in ways that make the associated benchmarking procedures effectively non-reproducible. As a result, even the fundamental property of reproducibility is not satisfied in most CT benchmarking studies.

By highlighting these limitations and analyzing prior work, we developed a data model and formalized the data preparation and quality assessment schemes currently used in practice. We then extended these schemes to support the preparation of datasets applicable across a broader range of tasks. Crucially, the extended schemes substantially enhance reproducibility in benchmarking, thereby advancing CT benchmarking to a new methodological level.

Building on these schemes, we formulated a set of essential components and conditions forming the foundation for reliable and reproducible data preparation and metric comparison. We strongly encourage researchers involved in CT data curation and method evaluation to integrate these components and conditions into their workflows. In particular, we emphasize the importance of fully exploiting the potential of vCT, which provides highly realistic data along with analytically computable phantoms. Surprisingly, despite its clear advantages, this approach is still not widely adopted in current research. Instead, researchers often rely on practices such as modifying already acquired real sinograms while defining the ground truth as a high-quality reconstruction.

Many of the proposed components and conditions build upon practices already well established in other domains, particularly in ML and DL, where benchmarking methodologies are far more mature. This work represents an initial step toward the establishment of a standard for reproducible benchmarking in CT, aiming to align the field with broader scientific practices and to foster more transparent, rigorous, and comparable research outcomes.

## Figures and Tables

**Table 1:** Levels of research reproducibility (types of validation studies), adapted from [8] and extended by level L0.

Id	Type	Description
L0	non-repeatability	different runs give different results with the same experimental setup
L1	repeatability	the same team can obtain consistent results using the same experimental setup
L2	reproducibility	external researchers are able to validate the correctness of the original experiment’s findings by following the documented experimental setup
L3	direct replicability	an independent team intentionally varies the implementation of the experiment—while keeping the hypothesis and experimental design consistent with the original study—to verify the results
L4	conceptual replicability	an independent team tests the same hypothesis through a fundamentally new experimental approach

**Table 2:** Publicly available volumetric vCT datasets for benchmarking.

Dataset	Year	Data	CT Scheme	Detector	Target Data	Variation	Spectrum	BHC	GT
LoDoPaB-CT [59]	2019	[60]	parallel	line	slice	dose	mono	–	normal dose reconstruction
SynDeepLesion [61,62]	202? ^1^	[63] ^2^	fan	line	slice	metal	poly	no [61] yes [64]	metal-free reconstruction
AAPM TrueCT [65]	2022	ask ^3^	helical	curved	volume	dose	poly	yes	effective energy phantom equivalent
AAPM CT-MAR [66]	2023	[67] ^4^	fan (cone)	line (curved)	slice	metal	poly	yes	metal-free reconstruction
ICASSP CBCT [68]	2024	[69]	cone	flat	volume	dose	mono	–	normal dose reconstruction

^1^—The exact timing of data release is difficult to determine. ^2^—Dataset hosted on China cloud storage; challenging to download or reproduce. ^3^—Available upon request, not independently verified. ^4^—Partially available, generation scripts provided.

**Table 3:** Data transformations and their degree of determinism affecting reproducibility.

Id	Transition	Level	Description
H2I(	H→I	det	windowing $i_{\left[c,w\right]}=H2I_{\left[w\right]}\left(h_{\left[c\right]}\right)$
H2R(	H→R	det|rnd	dequantifying tissue density from HU to LAC $r_{\left[c,e\right]}=H2R_{\left[e\right]}\left(h_{\left[c\right]}\right)$
P2R(	P→R	var	reconstruction $r_{\left[c,e\right]}=P2R_{\left[e\right]}\left(p\right)$
R2H(	R→H	det	quantifying tissue density in HU units $h_{\left[c\right]}=R2H\left(r_{\left[c,e\right]}\right)$
R2S(	R→S	var	reconstruction segmentation $s_{\left[c,\bar{m}\right]}=R2S_{\left[\bar{m}\right]}\left(r_{\left[c,e\right]}\right)$
R2V(	R→V	var	reconstruction vectorization $v_{\left[c\right]}=R2V\left(r_{\left[c,e\right]}\right)$
R2W(	R→W	var	material separation $w_{\left[c,\bar{m}\right]}=R2W_{\left[\bar{m}\right]}\left(r_{\left[c,e\right]}\right)$
S2V(	S→V	var	vectorization of a segmentation mask $v_{\left[c\right]}=S2V\left(s_{\left[c\right]}\right)$
S2W(	S→W	det	material mapping $w_{\left[c,\bar{m}\right]}=S2W\left(s_{\left[c,\bar{m}\right]}\right)$
V2P(	V→P	rnd	vCT projection of a vector phantom
V2S(	V→S	det	voxelization of a vector model into a segmentation mask $s_{\left[c,\bar{m}\right]}=V2S_{\left[c,\bar{m}\right]}\left(v\right)$
V2W(	V→W	det	voxelization of a vector model into a material weight mask $w_{\left[c,\bar{m}\right]}=V2W_{\left[c,\bar{m}\right]}\left(v\right)$
W2P(	W→P	var|rnd	vCT projection of a voxel phantom
W2R(	W→R	det	material-based LAC synthesis $r_{\left[c,e\right]}=W2R_{\left[e\right]}(w_{\left[c,\bar{m}\right]})$
W2S(	W→S	var	segmentation/binarization of material weights
BHC(	P→P	var	correction of sinograms for BHC
P2P(	P→P	var	correction of sinograms, including BHC, ring suppression, and others
NOI(	P→P	rnd	noise contamination of sinograms (low photon counting)
DSS(	P→P	det	deterministic sinogram subsampling (sparse, limited angle and any-time CT)
RSS(	P→P	rnd	randomized sinogram subsampling (any-time CT)
DEN(	X→X X∈{P,R,H,I}	var	denoising
RES(	X→X X∈{R,H,I,S,W}	var	volume-based data resampling $x_{\left[c_{2}\right]}={\mathrm{RES}}_{\left[c_{2},c_{1}\right]}\left(x_{\left[c_{1}\right]}\right)$ betweem CSs $c_{1}$ and $c_{2}$
FOV(	X→S X∈{R,H,I}	det	binary FOV mask for reconstruction $f_{\left[c\right]}^{FOV}=\mathrm{FOV}\left(x_{\left[c\right]}\right)$

**Table 4:** Material property values (XCIST database, .ncat files) modeled in XCIST for energies 80kEv and 120kEv. The values of $\mu^{e_{ref}}$ are given in ${\mathrm{cm}}^{-1}$.

Material	$\mu^{80\;\mathrm{kEv}}$	$\mu^{120\;\mathrm{kEv}}$	${\mathrm{HU}}_{\mathrm{air}}^{80\;\mathrm{kEv}}$	${\mathrm{HU}}_{\mathrm{air}}^{120\;\mathrm{kEv}}$	${\mathrm{HU}}_{\mathrm{vac}}^{80\;\mathrm{kEv}}$	${\mathrm{HU}}_{\mathrm{vac}}^{120\;\mathrm{kEv}}$
air	0.000200	0.000154	−1000.0	−1000.0	−998.9	−998.9
lung	0.047451	0.036553	−742.3	−742.9	−741.5	−742.1
water	0.183566	0.141741	0.0	0.0	0.0	0.0
pancreas	0.189531	0.146516	32.5	33.7	32.5	33.7
skull	0.341355	0.222230	860.5	568.5	859.6	567.9
aluminum	0.544019	0.343439	1965.8	1424.6	1963.6	1423.0
titanium	1.835591	0.640721	9009.5	3524.2	8999.6	3520.4

**Table 5:** Classification of quality assessment functions by input data (TF—task full reference, TM—task model reference, TD—task-specific downstream).

Class Id	*r*	*s* *v*	$r^{\mathrm{ref}}$	$s^{\mathrm{ref}}$ $v^{\mathrm{ref}}$ $f^{\mathrm{VOI}}$	Examples	Description
QTF(	+		+	+	SNR∗,RFS ^1^	task-specific with image reference
QTM(	+			+	CNR∗,NPS,MTF	task-specific without image reference
QTD(		+		+	DC ^2^,HD ^3^$,d^{\prime }$	task-specific downstream application
QFR(	+		+		$PSNR,SSIM,VIF,HaarPsi_{MED}$	generic with image reference (FR-IQA)
QNR(	+				BRISQUE ^4^, CT-NR-IQA ^5^	no reference (NR-IQA)

∗—regional (in *VOI*) evaluation. ^1^—radiomic feature similarity (*RFS*) [32]. ^2^—Dice Coefficient (*DC*) for segmentation mask. ^3^—Hausdorff Distance (*HD*) for segmentation mask. ^4^—Blind/Referenceless Image Spatial Quality Evaluator (*BRISQUE*) [94]. ^5^—CNN-based CT image NR-IQA [95]. The plus sign (+) indicates the types of data that are used for quality assessment via the corresponding class of quality assessment functions.

**Table 6:** Types of CT downstream tasks.

*n*	GT	Description
1		classification
2	*s*	segmentation (including semantic segmentation such as material decomposition, as well as instance and panoptic segmentation)
3	*v*	vectorization
4	*v*	object detection
5		direct estimation of volume characteristics (without explicit use of items 1–4)
6		prediction (e.g., survival prediction in medicine)
7	*r*	image harmonization and normalization (i.e., rescaling to a standard spatial resolution and intensity range, including super-resolution techniques)
8	$g_{\left[c_{i},c_{j}\right]}$	registration
8.1		CT image registration
8.2		limited-overlap region registration (image stitching)
8.3		multi-modal (tomographic) image registration
8.4		multi-scale (tomographic) image registration
8.5		vector-model to tomographic image registration

**Table 7:** Essential components and conditions for reproducible CT benchmarking, enhancing the reproducibility of reconstruction algorithm quality assessment.

n	Level	xCT Section 5.6	vCT Section 5.9	Description
1	L1	+	+	deterministic implementation (CPU vs GPU, libraries, etc.)
2	L1	+	+	specified sinogram subsampling
3	L1		+	fixed random seed in the simulation of stochastic processes
4	L2	+	+	dataset includes data (sinograms + GT) of the calibration and validation objects
5	L2	+	+	dataset includes masks $f^{FOV}$
6	L2	+	+	dataset includes masks $f^{VOI}$
7	L2	+	+	dataset includes raw sinograms $p^{raw}$ (raw scan data) as input into the sinogram preprocessing stage $p_{\left[e\right]}=P2P\left(p^{raw}\right)$
8	L2	+	+	dataset includes intermediate sinograms obtained during the sinogram preprocessing stage $p_{\left[e\right]}=P2P\left(p^{raw}\right)$
9	L2	+	+	dataset includes reconstruction-ready sinograms $p_{\left[e\right]}$ as output of the sinogram preprocessing stage $p_{\left[e\right]}=P2P\left(p^{raw}\right)$
10	L2	+	+	dataset includes volumes reconstructed using a baseline method (specified method with fixed parameters)
11	L2	+	+	specified method with fixed parameters for dequantifying tissue density H2R
12	L2	+	+	specified method with fixed parameters for quantifying tissue density R2H
13	L2	+	+	specified method with fixed parameters for resampling RES
14	L2	+	+	quality is assessed using functions with a specified implementation and fixed parameters (including the required normalization of reconstruction value ranges)
15	L2	+	+	quality is assessed in the specified fixed CS
16	L2	+	+	quality is assessed along with the computation of uncertainty measures for the obtained estimates
17	L2	+	+	quality is assessed within the specified $f^{FOV}$
18	L2	+	+	specified method with fixed parameters for sinogram preprocessing $p_{\left[e\right]}=P2P\left(p^{raw}\right)$, including BHC
19	L2		+	dataset includes analytical GT $r_{\left[\mathrm{tgt},e\right]}^{syn}$ for the reconstructions
20	L2		+	quality is assessed using analytical GT $r_{\left[\mathrm{tgt},e\right]}^{syn}$
21	L2		+	vCT specified method with fixed material decomposition parameters R2W|H2W used to design phantoms
22	L2		+	vCT specified method with fixed parameters for noise modeling
23	L2		+	vCT specified method for simulating the polychromatic spectrum of the probing radiation with determined parameters (including filters)
24	L2		+	vCT specified method with fixed parameters for scattering modeling
25	L2		+	vCT specified method with fixed parameters for X-ray absorption modeling (with material-specific reference LAC values also provided)
26	L2		+	vCT specified method with fixed parameters for modeling projection registration
27	L3	+	+	dataset includes composite objects with varying configurations
28	L3	+	+	dataset includes objects in multiple geometric positions, together with information on these positions (intended for registration)
29	L3	+	+	dataset includes objects scanned under different acquisition protocols
30	L3	+	+	dataset includes objects equipped with reference features (intended for registration tasks)
31	L3	+	+	dataset includes digital models of objects (digital twins), either segmentation-based and/or vector-based
32	L3	+	+	quality is assessed using various task-specific ranking methods
33	L3	+	+	quality assessment functions’ sensitivity to the selected reference reconstruction method, P2R is examined
34	L3		+	published pipeline for benchmark data generation

## Data Availability

The original contributions presented in this study are included in the article. Further inquiries can be directed to the corresponding authors.

## Abbreviations

The following abbreviations are used in this manuscript:

BH	beam hardening
BHC	beam hardening correction
CAD	computer-aided design
CNN	convolutional neural network
CNR	contrast-to-noise ratio
conCT	conveyor computed tomography
CS	coordinate system
CT	computed tomography
DL	deep learning
ESF	edge spread function
FID	Fréchet inception distance
FOV	field of view
FR-IQA	full-reference image quality assessment
GCS	global coordinate system
HU	Hounsfield units
indCT	industrial computed tomography
IQM	image quality metric
labCT	laboratory computed tomography
LAC	linear attenuation coefficient
LDCT	low-dose computed tomography
LDR	low-dose reconstruction
LSF	line spread function
MAR	metal artifact reduction
medCT	medical computed tomography
ML	machine learning
MTF	modulation transfer function
MRI	magnetic resonance imaging
NPS	noise power spectrum
NR-IQA	no-reference image quality assessment
PET	positron emission tomography
OCS	object’s coordinate system
PSF	point spread function
PSNR	peak signal-to-noise ratio
RFS	radiomic feature similarity
RIC	ring artifact correction
RMSE	root mean square error
ROI	region(s) of interest
RQA	reference-based quality assessment
SAT	subjective assessment techniques
SSIM	structural similarity index measure
TSM	triangulated surface mesh
TTF	task transfer function
vCT	virtual computed tomography
VIF	visual information fidelity
VOI	volume(s) of interest
WMM	weighted material map
xCT	X-ray computed tomography

## References

[1] Weber L.M., Saelens W., Cannoodt R., Soneson C., Hapfelmeier A., Gardner P.P., Boulesteix A.L., Saeys Y., Robinson M.D. (2019). Essential guidelines for computational method benchmarking. Genome Biol..

[2] Ioannidis J.P. (2005). Why most published research findings are false. PLoS Med..

[3] Shimron E., Tamir J.I., Wang K., Lustig M. (2022). Implicit data crimes: Machine learning bias arising from misuse of public data. Proc. Natl. Acad. Sci. USA.

[4] Maier-Hein L., Reinke A., Godau P., Tizabi M.D., Buettner F., Christodoulou E., Glocker B., Isensee F., Kleesiek J., Kozubek M. (2024). Metrics reloaded: Recommendations for image analysis validation. Nat. Methods.

[5] Polevoy D.V., Kazimirov D.D., Mehova M.S., Gilmanov M.I., Pang X. (2025). X-Ray Computed Tomography Traps and Challenges for Deep Learning Scientist. Proceedings of the SPRA 2024.

[6] Gundersen O.E. (2021). The fundamental principles of reproducibility. Philos. Trans. R. Soc. A.

[7] Semmelrock H., Ross-Hellauer T., Kopeinik S., Theiler D., Haberl A., Thalmann S., Kowald D. (2025). Reproducibility in machine-learning-based research: Overview, barriers, and drivers. AI Mag..

[8] Desai A., Abdelhamid M., Padalkar N.R. (2025). What is reproducibility in artificial intelligence and machine learning research?. AI Mag..

[9] Chikin P.S., Soldatova Z.V., Ingacheva A.S., Polevoy D.V. (2024). Virtual data generation pipeline for the analysis of computed tomography methods. Tr. ISA RAN (Proc. ISA RAS).

[10] Arlazarov V.L., Nikolaev D.P., Arlazarov V.V., Chukalina M.V. (2021). X-ray Tomography: The Way from Layer-by-layer Radiography to Computed Tomography. Comput. Opt..

[11] Bellens S., Guerrero P., Vandewalle P., Dewulf W. (2024). Machine learning in industrial X-ray computed tomography–a review. CIRP J. Manuf. Sci. Technol..

[12] Renard F., Guedria S., Palma N.D., Vuillerme N. (2020). Variability and reproducibility in deep learning for medical image segmentation. Sci. Rep..

[13] Polevoy D., Gilmanov M., Kazimirov D., Chukalina M., Ingacheva A., Kulagin P., Nikolaev D. (2023). Tomographic reconstruction: General approach to fast back-projection algorithms. Mathematics.

[14] Polevoy D.V., Kazimirov D.D., Chukalina M.V., Nikolaev D.P. (2024). Complexity-Preserving Transposition of Summing Algorithms: A Data Flow Graph Approach. Probl. Inf. Transm..

[15] Levine Z.H., Peskin A.P., Holmgren A.D., Garboczi E.J. (2018). Preliminary X-ray CT investigation to link Hounsfield unit measurements with the International System of Units (SI). PLoS ONE.

[16] Buzug T.M. (2011). Computed tomography. Springer Handbook of Medical Technology.

[17] Ingacheva A.S., Chukalina M.V. (2019). Polychromatic CT Data Improvement with One-Parameter Power Correction. Math. Probl. Eng..

[18] Gilmanov M., Kirkicha A., Ingacheva A., Chukalina M., Nikolaev D., Pang X. (2025). Stability of artificial intelligence methods in computed tomography metal artifacts reduction task. Proceedings of the SPRA 2024.

[19] Shutov M., Gilmanov M., Polevoy D., Buzmakov A., Ingacheva A., Chukalina M., Nikolaev D., Osten W., Nikolaev D., Debayle J. (2024). CT metal artifacts simulation under x-ray total absorption. Proceedings of the ICMV 2023.

[20] Zhao W., Vernekohl D., Zhu J., Wang L., Xing L. (2016). A model-based scatter artifacts correction for cone beam CT. Med. Phys..

[21] Yamaev A.V., Chukalina M.V., Nikolaev D.P., Sheshkus A.V., Chulichkov A.I. (2021). Neural Network for Data Preprocessing in Computed Tomography. Autom Remote Control.

[22] Yamaev A.V., Chukalina M.V., Nikolaev D.P., Sheshkus A.V., Chulichkov A.I. (2021). Lightweight Denoising Filtering Neural Network For FBP Algorithm. Proceedings of the ICMV 2020.

[23] Yamaev A.V., Chukalina M.V., Nikolaev D.P., Kochiev L.G., Chulichkov A.I. (2022). Neural network regularization in the problem of few-view computed tomography. Comput. Opt..

[24] Buzmakov A., Zolotov D., Chukalina M., Ingacheva A., Asadchikov V., Nikolaev D., Krivonosov Y., Dyachkova I., Bukreeva I. (2021). Iterative Reconstruction of Incomplete Tomography Data: Application Cases. Proceedings of the ICMV 2020.

[25] Gilmanov M.I., Bulatov K.B., Bugai O.A., Ingacheva A.S., Chukalina M.V., Nikolaev D.P., Arlazarov V.V. (2024). Applicability and potential of monitored reconstruction in computed tomography. PLoS ONE.

[26] Bulatov K.B., Ingacheva A.S., Gilmanov M.I., Kutukova K., Soldatova Z.V., Buzmakov A.V., Chukalina M.V., Zschech E., Arlazarov V.V. (2023). Towards monitored tomographic reconstruction: Algorithm-dependence and convergence. Comput. Opt..

[27] Ingacheva A., Bulatov K., Soldatova Z., Kutukova K., Chukalina M., Nikolaev D., Arlazarov V., Zschech E. (2022). Comparison convergence of the reconstruction algorithms for monitored tomography on synthetic dataset. Synchrotron and Free Electron Laser Radiation: Generation and Application (SFR-2022).

[28] Kazimirov D., Polevoy D., Ingacheva A., Chukalina M., Nikolaev D. (2024). Adaptive automated sinogram normalization for ring artifacts suppression in CT. Opt. Express.

[29] Kazimirov D., Ingacheva A., Buzmakov A., Chukalina M., Nikolaev D., Osten W., Nikolaev D., Debayle J. (2024). Robust Automatic Rotation Axis Alignment Mean Projection Image Method in Cone-Beam and Parallel-Beam CT. Proceedings of the ICMV 2023.

[30] Kazimirov D., Ingacheva A., Buzmakov A., Marina C., Dmitry N., Osten W., Nikolaev D., Zhou J. (2023). Mean projection image application to the automatic rotation axis alignment in cone-beam CT. Proceedings of the ICMV 2022.

[31] Kyme A.Z., Fulton R.R. (2021). Motion estimation and correction in SPECT, PET and CT. Phys. Med. Biol..

[32] Eulig E., Ommer B., Kachelrieß M. (2024). Benchmarking deep learning-based low-dose CT image denoising algorithms. Med. Phys..

[33] Fedorov A., Longabaugh W.J., Pot D., Clunie D.A., Pieper S.D., Gibbs D.L., Bridge C., Herrmann M.D., Homeyer A., Lewis R. (2023). National cancer institute imaging data commons: Toward transparency, reproducibility, and scalability in imaging artificial intelligence. Radiographics.

[34] Elfer K., Gardecki E., Garcia V., Ly A., Hytopoulos E., Wen S., Hanna M.G., Peeters D.J., Saltz J., Ehinger A. (2024). Reproducible reporting of the collection and evaluation of annotations for artificial intelligence models. Mod. Pathol..

[35] Samala R.K., Gallas B.D., Zamzmi G., Juluru K., Khan A., Bahr C., Ochs R., Carranza D., Granstedt J., Margerrison E. (2025). Medical Imaging Data Strategies for Catalyzing AI Medical Device Innovation. J. Imaging Inform. Med..

[36] Zwanenburg A., Vallières M., Abdalah M.A., Aerts H.J., Andrearczyk V., Apte A., Ashrafinia S., Bakas S., Beukinga R.J., Boellaard R. (2020). The image biomarker standardization initiative: Standardized quantitative radiomics for high-throughput image-based phenotyping. Radiology.

[37] Kuhl C.K., Truhn D. (2020). The long route to standardized radiomics: Unraveling the knot from the end. Radiology.

[38] Kocak B., Baessler B., Bakas S., Cuocolo R., Fedorov A., Maier-Hein L., Mercaldo N., Müller H., Orlhac F., Pinto dos Santos D. (2023). CheckList for EvaluAtion of Radiomics research (CLEAR): A step-by-step reporting guideline for authors and reviewers endorsed by ESR and EuSoMII. Insights Imaging.

[39] McCollough C.H., Bartley A.C., Carter R.E., Chen B., Drees T.A., Edwards P., Holmes III D.R., Huang A.E., Khan F., Leng S. (2017). Low-dose CT for the detection and classification of metastatic liver lesions: Results of the 2016 Low Dose CT Grand Challenge. Med. Phys..

[40] Moen T.R., Chen B., Holmes III D.R., Duan X., Yu Z., Yu L., Leng S., Fletcher J.G., McCollough C.H. (2021). Low-dose CT image and projection dataset. Med. Phys..

[41] Kiss M.B., Coban S.B., Batenburg K.J., van Leeuwen T., Lucka F. (2023). 2DeteCT-A large 2D expandable, trainable, experimental Computed Tomography dataset for machine learning. Sci. Data.

[42] Kazantsev D., Beveridge L., Shanmugasundar V., Magdysyuk O. (2024). Conditional generative adversarial networks for stripe artefact removal in high-resolution x-ray tomography. Tomogr. Mater. Struct..

[43] Vo N.T., Atwood R.C., Drakopoulos M. (2018). Superior techniques for eliminating ring artifacts in X-ray micro-tomography. Opt. Express.

[44] Vo N.T., Atwood R.C., Drakopoulos M. (2018). Tomographic data for testing, demonstrating, and developing methods of removing ring artifacts. Zenodo.

[45] Kudo H., Suzuki T., Rashed E.A. (2013). Image reconstruction for sparse-view CT and interior CT—introduction to compressed sensing and differentiated backprojection. Quant. Imaging Med. Surg..

[46] Kiss M.B., Biguri A., Shumaylov Z., Sherry F., Batenburg K.J., Schönlieb C.B., Lucka F. (2025). Benchmarking learned algorithms for computed tomography image reconstruction tasks. Appl. Math. Mod. Challenges.

[47] Guo J., Qi H., Xu Y., Chen Z., Li S., Zhou L. (2016). Iterative Image Reconstruction for Limited-Angle CT Using Optimized Initial Image. Comput. Math. Methods Med..

[48] Leuschner J., Schmidt M., Ganguly P.S., Andriiashen V., Coban S.B., Denker A., Bauer D., Hadjifaradji A., Batenburg K.J., Maass P. (2021). Quantitative comparison of deep learning-based image reconstruction methods for low-dose and sparse-angle CT applications. J. Imaging.

[49] Usui K., Kamiyama S., Arita A., Ogawa K., Sakamoto H., Sakano Y., Kyogoku S., Daida H. (2024). Reducing image artifacts in sparse projection CT using conditional generative adversarial networks. Sci. Rep..

[50] Piccolomini E.L., Evangelista D., Morotti E. (2025). Deep Guess acceleration for explainable image reconstruction in sparse-view CT. Comput. Med Imaging Graph..

[51] Bulatov K., Chukalina M., Buzmakov A., Nikolaev D., Arlazarov V.V. (2020). Monitored Reconstruction: Computed Tomography as an Anytime Algorithm. IEEE Access.

[52] Yamaev A.V. (2024). Monitored reconstruction improved by post-processing neural network. Comput. Opt..

[53] Yu L., Shiung M., Jondal D., McCollough C.H. (2012). Development and validation of a practical lower-dose-simulation tool for optimizing computed tomography scan protocols. J. Comput. Assist. Tomogr..

[54] Žabić S., Wang Q., Morton T., Brown K.M. (2013). A low dose simulation tool for CT systems with energy integrating detectors. Med. Phys..

[55] Zeng D., Huang J., Bian Z., Niu S., Zhang H., Feng Q., Liang Z., Ma J. (2015). A simple low-dose X-ray CT simulation from high-dose scan. IEEE Trans. Nucl. Sci..

[56] Gibson N.M., Lee A., Bencsik M. (2024). A practical method to simulate realistic reduced-exposure CT images by the addition of computationally generated noise. Radiol. Phys. Technol..

[57] Yadav A., Welland S.H., Hoffman J.M., Kim H., Brown M.S., Prosper A.E., Aberle D.R., McNitt-Gray M.F., Hsu W. (2025). A comparative analysis of image harmonization techniques in mitigating differences in CT acquisition and reconstruction. Phys. Med. Biol..

[58] Kiss M.B., Biguri A., Schönlieb C.B., Batenburg K.J., Lucka F. (2024). Learned denoising with simulated and experimental low-dose CT data. arXiv.

[59] Leuschner J., Schmidt M., Baguer D.O., Maass P. (2021). LoDoPaB-CT, a benchmark dataset for low-dose computed tomography reconstruction. Sci. Data.

[60] Leuschner J., Schmidt M., Otero Baguer D. (2019). LoDoPaB-CT Dataset. https://zenodo.org/records/3384092.

[61] Zhang Y., Yu H. (2018). Convolutional neural network based metal artifact reduction in X-ray computed tomography. IEEE Trans. Med Imaging.

[62] Yu L., Zhang Z., Li X., Xing L. (2020). Deep sinogram completion with image prior for metal artifact reduction in CT images. IEEE Trans. Med Imaging.

[63] Wang H. (2023). SynDeepLesion. https://github.com/hongwang01/SynDeepLesion.

[64] Sakamoto M., Hiasa Y., Otake Y., Takao M., Suzuki Y., Sugano N., Sato Y. (2019). Automated segmentation of hip and thigh muscles in metal artifact contaminated CT using CNN. Proceedings of the International Forum on Medical Imaging in Asia 2019.

[65] Abadi E., Segars W.P., Felice N., Sotoudeh-Paima S., Hoffman E.A., Wang X., Wang W., Clark D., Ye S., Jadick G. (2025). AAPM Truth-based CT (TrueCT) reconstruction grand challenge. Med. Phys..

[66] American Association of Physicists in Medicine (2021). AAPM CT-MAR Grand Challenge. https://www.aapm.org/GrandChallenge/CT-MAR/.

[67] (2025). AAPM CT-MAR Challenge. https://github.com/xcist/example/tree/main/AAPM_datachallenge.

[68] Biguri A., Mukherjee S., Zhao X., Liu X., Wang X., Yang R., Du Y., Peng Y., Brudfors M., Graham M. (2024). Advancing the Frontiers of Deep Learning for Low-Dose 3D Cone-Beam CT Reconstruction. TechRxiv.

[69] Biguri A. (2024). Test dataset for the ICASSP-2024 3D-CBCT challenge (Part 1). Zenodo.

[70] Herman G.T. (1979). Correction for beam hardening in computed tomography. Phys. Med. Biol..

[71] Wirgin A. (2004). The Inverse Crime. arXiv.

[72] Lewitt R.M. (2005). Reconstruction algorithms: Transform methods. Proc. IEEE.

[73] Wu M., FitzGerald P., Zhang J., Segars W.P., Yu H., Xu Y., De Man B. (2022). XCIST—an open access x-ray/CT simulation toolkit. Phys. Med. Biol..

[74] Samei E., Bakalyar D., Boedeker K.L., Brady S., Fan J., Leng S., Myers K.J., Popescu L.M., Ramirez Giraldo J.C., Ranallo F. (2019). Performance evaluation of computed tomography systems: Summary of AAPM Task Group 233. Med. Phys..

[75] Xun S., Li Q., Liu X., Huang P., Zhai G., Sun Y., Wu M., Tan T. (2025). Charting the path forward: CT image quality assessment-an in-depth review. J. King Saud Univ. Comput. Inf. Sci..

[76] Kim W., Jeon S.Y., Byun G., Yoo H., Choi J.H. (2024). A systematic review of deep learning-based denoising for low-dose computed tomography from a perceptual quality perspective. Biomed. Eng. Lett..

[77] Herath H., Herath H., Madusanka N., Lee B.I. (2025). A Systematic Review of Medical Image Quality Assessment. J. Imaging.

[78] Sheikh H.R., Bovik A.C. (2006). Image information and visual quality. IEEE Trans. Image Process..

[79] Heusel M., Ramsauer H., Unterthiner T., Nessler B., Hochreiter S. (2017). Gans trained by a two time-scale update rule converge to a local nash equilibrium. Adv. Neural Inf. Process. Syst..

[80] Karner C., Gröhl J., Selby I., Babar J., Beckford J., Else T.R., Sadler T.J., Shahipasand S., Thavakumar A., Roberts M. Parameter choices in HaarPSI for IQA with medical images. Proceedings of the 2025 IEEE International Symposium on Biomedical Imaging (ISBI).

[81] Verdun F., Racine D., Ott J., Tapiovaara M., Toroi P., Bochud F., Veldkamp W., Schegerer A., Bouwman R., Giron I.H. (2015). Image quality in CT: From physical measurements to model observers. Phys. Medica.

[82] Ohashi K., Nagatani Y., Yoshigoe M., Iwai K., Tsuchiya K., Hino A., Kida Y., Yamazaki A., Ishida T. (2023). Applicability Evaluation of Full-Reference Image Quality Assessment Methods for Computed Tomography Images. J. Digit. Imaging.

[83] Breger A., Karner C., Selby I., Gröhl J., Dittmer S., Lilley E., Babar J., Beckford J., Else T.R., Sadler T.J. (2024). A study on the adequacy of common IQA measures for medical images. arXiv.

[84] Lee W., Wagner F., Galdran A., Shi Y., Xia W., Wang G., Mou X., Ahamed M.A., Imran A.A.Z., Oh J.E. (2025). Low-dose computed tomography perceptual image quality assessment. Med. Image Anal..

[85] Nikolaev D., Buzmakov A., Chukalina M., Yakimchuk I., Gladkov A., Ingacheva A., Bernstein A.V., Olaru A., Zhou J. (2017). CT Image Quality Assessment Based on Morphometric Analysis of Artifacts. Proceedings of the ICRMV 2016.

[86] Ingacheva A., Chukalina M., Buzmakov A., Nikolaev D., Osten W., Nikolaev D., Zhou J. (2020). Method for numeric estimation of Cupping effect on CT images. Proceedings of the ICMV 2019.

[87] Ingacheva A.S., Tropin D.V., Chukalina M.V., Nikolaev D.P. (2021). Blind CT images quality assessment of cupping artifacts. Proceedings of the ICMV 2020.

[88] Tunissen S.A.M., Oostveen L.J., Moriakov N., Teuwen J., Michielsen K., Smit E.J., Sechopoulos I. (2023). Development, validation, and simplification of a scanner-specific CT simulator. Med. Phys..

[89] Alsaihati N., Solomon J., McCrum E., Samei E. (2025). Development, validation, and application of a generic image-based noise addition method for simulating reduced dose computed tomography images. Med. Phys..

[90] Tan Y., Kiekens K., Welkenhuyzen F., Angel J., De Chiffre L., Kruth J.P., Dewulf W. (2014). Simulation-aided investigation of beam hardening induced errors in CT dimensional metrology. Meas. Sci. Technol..

[91] Kronfeld A., Rose P., Baumgart J., Brockmann C., Othman A.E., Schweizer B., Brockmann M.A. (2024). Quantitative multi-energy micro-CT: A simulation and phantom study for simultaneous imaging of four different contrast materials using an energy integrating detector. Heliyon.

[92] Fan Y., Pack J., De Man B. (2022). A virtual imaging trial framework to study cardiac CT blooming artifacts. Proceedings of the 7th International Conference on Image Formation in X-Ray Computed Tomography.

[93] Paramonov P., Francken N., Renders J., Iuso D., Elberfeld T., Beenhouwer J.D., Sijbers J. (2024). CAD-ASTRA: A versatile and efficient mesh projector for X-ray tomography with the ASTRA-toolbox. Opt. Express.

[94] Mittal A., Moorthy A.K., Bovik A.C. (2012). No-reference image quality assessment in the spatial domain. IEEE Trans. Image Process..

[95] Ohashi K., Nagatani Y., Yamazaki A., Yoshigoe M., Iwai K., Uemura R., Shimomura M., Tanimura K., Ishida T. (2025). Development of a No-Reference CT Image Quality Assessment Method Using RadImageNet Pre-trained Deep Learning Models. J. Imaging Inform. Med..

[96] Wang H., Li Y., Zhang H., Chen J., Ma K., Meng D., Zheng Y. (2021). InDuDoNet: An Interpretable Dual Domain Network for CT Metal Artifact Reduction. International Conference on Medical Image Computing and Computer-Assisted Intervention.

[97] Kyriakou Y., Meyer E., Prell D., Kachelrieß M. (2010). Empirical beam hardening correction (EBHC) for CT. Med. Phys..

[98] Madesta F., Sentker T., Rohling C., Gauer T., Schmitz R., Werner R. (2024). Monte Carlo-based simulation of virtual 3 and 4-dimensional cone-beam computed tomography from computed tomography images: An end-to-end framework and a deep learning-based speedup strategy. Phys. Imaging Radiat. Oncol..

[99] Smolin A., Yamaev A., Ingacheva A., Shevtsova T., Polevoy D., Chukalina M., Nikolaev D., Arlazarov V. (2022). Reprojection-based numerical measure of robustness for CT reconstruction neural networks algorithms. Mathematics.

[100] Karius A., Bert C. (2022). QAMaster: A new software framework for phantom-based computed tomography quality assurance. J. Appl. Clin. Med. Phys..

[101] Zhang J., Wu M., FitzGerald P., Araujo S., De Man B. (2024). Development and tuning of models for accurate simulation of CT spatial resolution using CatSim. Phys. Med. Biol..

